# Functional genomic dissection and prediction of body size traits in pigs

**DOI:** 10.1186/s12711-026-01078-1

**Published:** 2026-08-16

**Authors:** Naibiao Yu, Dengshuai Cui, Lei Xie, Xi Tang, Sanya Xiong, Yang Zhang, Ruiqiu He, Longyun Li, Shijun Xiao, Yuanmei Guo

**Affiliations:** National Key Laboratory for Swine Genetic Improvement and Germplasm Innovation, Ministry of Science and Technology of China, Jiangxi Agricultural University, Nanchang, 330045 China

## Abstract

**Background:**

Body Size traits, particularly body weight (BW) and body mass index (BMI) at slaughter age, determine the meat yield and productivity of pigs. These phenotypes are shaped by numerous small-effect polygenes and regulated mostly by non-coding variants. Although genome-wide association studies (GWAS) have identified several loci and candidate functional variants, the regulatory mechanisms and causative genes of most traits remain uncharacterized, which limits the effectiveness of genomic prediction (GP). The purpose of this study was to bridge the gap between association studies and GP by integrating regulatory genomics into the GP framework to enhance prediction accuracy for body size traits.

**Results:**

Using imputation-based GWAS in 1226 Shanxia Long Black pigs, multiple genome-wide significant loci were identified to be associated with BW and BMI. Linkage disequilibrium (LD) analysis, SuSiE fine-mapping, and regulatory modeling with *Basenji* deep-learning predictions refined these associations to 10 quantitative trait loci (QTLs) with 45 high-confidence candidate functional variants. Integration of chromatin-state annotations and high-throughput chromosome conformation capture (Hi-C) data revealed receptor tissue regulatory architectures; BW-associated variants on *Sus scrofa* chromosome 2 (SSC2) were enriched for brain regulatory regions, whereas BMI-associated loci showed enhancer activity across adipose, brain, and liver tissues. Multi-omics analyses converged on *ZER1*, *KLHL29*, and *HAO1* as high-confidence candidate genes, while *OR2T27* was a putative candidate on SSC2. In *Basenji* prediction, several specific candidate variants were identified as a liver enhancer. Incorporating these top-prioritized functional variants into genomic prediction models, GP yielded up to 21% gains in accuracy.

**Conclusions:**

This study dissects the multi-tissue regulatory architecture, identifying functional variants, effector tissues, and target genes underlying porcine BW and BMI. By leveraging these biologically prioritized loci, we established a functionally informed GP framework that enhances prediction accuracy and biological interpretability simultaneously and also offered a scalable strategy for genetic improvement of complex traits in livestock.

**Supplementary Information:**

The online version contains supplementary material available at 10.1186/s12711-026-01078-1.

## Background

Genetic improvement of body size at slaughter age is a primary breeding objective in the swine industry [1], particularly for commercial breeds, because a larger body size at slaughter age directly benefits producers, processors, and consumers. Body weight (BW) and body mass index (BMI) are body size related traits and controlled by polygenes [2], with heritability distributed among many small-effect variants that predominantly map to non-coding regions of the genome.

With the substantial reduction in sequencing costs and the rapid increase in genetic marker density [3–5], GWAS has become the gold standard for mapping QTLs [6]. While numerous quantitative trait loci (QTLs) associated with porcine BW and BMI have been mapped [2, 7–10], identifying the causal mutations underlying these QTLs remains a formidable challenge [11]. To date, only a handful of causative genes and variants have been pinpointed. However, QTL mapping has provided critical insights into the genetic architectures of the complex traits in pigs, including obesity [12], vertebral number [13, 14], and thoracic vertebrae number [15–18], and have paved the way for precision breeding applications.

In modern livestock breeding, molecular markers facilitate the prediction of complex phenotypes via marker-assisted selection (MAS) and genomic prediction (GP) [19]. Genomic best linear unbiased prediction (GBLUP) operates effectively by assuming a uniform normal distribution for all marker effects [20, 21], but some Bayesian methods (e.g., BayesA, BayesB, and Bayesian LASSO) attempt to refine predictions by employing distinct prior distributions that explicitly accommodate varying marker-specific variances [20–23]. These approaches have improved accuracy across diverse traits [24–30], including for teat number in pigs [31]. Based on this foundation, including high-confidence functional variants into GP models could significantly enhance the prediction accuracy of estimated breeding values (EBV). However, the fine-scale functional regulatory architecture underlying BW and BMI in pigs is still largely unresolved.

To pinpoint the candidate functional variants of BW and BMI, an integrative framework combining experimental and computational approaches was carried out in this study. Firstly, GWAS was performed using imputed whole-genome sequence data to map BW and BMI loci. Then, LD and SuSiE fine-mapping were applied to prioritize causal variants [32]. To decode regulatory mechanisms, we integrated chromatin-state annotations, deep-learning-based variant effect predictions (*Basenji*) [33], and high-throughput chromosome conformation capture (Hi-C) data to link non-coding variants to their target genes. Finally, both empirical and simulated datasets were used to evaluate the performance of GP models that incorporate these functional priors as fixed effects. This study elucidates the multi-tissue regulatory architecture of porcine body size and demonstrates that leveraging functional genomics is a viable strategy to enhance genomic prediction accuracy.

## Methods

### Phenotype

A total of 1226 Shanxia Long Black pigs, including 77 barrows and 1149 gilts, were used in this study. All pigs were raised at the same farm of Jiangxi Shanxia Huaxi Pig Breeding and Raising Company Limited (Ganzhou, Jiangxi province, China) in six production batches [34], with ad libitum access to fresh water and corn-soybean meal feed, which contained 3100 kJ DE, 16% CP, and 0.78% lysine. Pigs were slaughtered at commercial abattoirs when their body weights were 90–125 kg at age of 210–240 d.

For each body size trait, measurements were taken by the same technician using identical equipment across six batches. BW and body length (BL) were recorded alive on-farm before the pigs were transported to commercial abattoirs, and BMI was the quotient of BW(kg) divided by the square of BL (m^2^). Statistical models were fitted using the *lm* function in R (v4.3.0), adjusting for covariates, including sex and batch.

### Genotyping, imputation, and quality control

The 1266 pigs were genotyped using the CC1 Porcine SNP50K BeadChip (Illumina, San Diego, CA, USA). After quality control with PLINK v1.90b7 [35] (call rate > 90%, minor allele frequency (MAF) > 5%, Hardy–Weinberg equilibrium (HWE) test *P* > 1 × 10^–5^), 34,103 single nucleotide polymorphisms (SNPs) remained, and all individuals were retained (call rate > 95%). Haplotypes were phased with SHAPEIT v5.1.1 [36] using our previously published pig reference panel [37], and genome-wide imputation was conducted using IMPUTE5 with default parameters [38]. The accuracy of imputation was evaluated using the internal cross-validation method in IMPUTE5. In brief, 30 SNPs were randomly selected, and their genotypes were masked for all pigs. Then, the genotypes of the masked SNPs were imputed utilizing the haplotypes from the reference panel. Finally, the concordance percentage and the squared correlation (*r*^2^) between the genotyped and imputed genotypes were calculated. The imputed data were of high quality (concordance rate = 99.16%; *r*^2^ = 0.89). After post-imputation filtering using PLINK v1.90b6.24 [35] (call rate > 99%, MAF > 1%, HWE *P* > 5 × 10^−6^), 14,609,355 SNPs were used for downstream analyses.

### GWAS and SNP-based heritability estimates

GEMMA software (version 0.98.1) [39] was used to conduct GWAS with the following linear mixed model (LMM):

1 * y = Xb + mg + Zu + e, *

where ***y***, ***b***, ***u***, and *e* are the vectors of phenotypes, fixed effects, polygenic effects, and random residuals, respectively, and ***X***, and ***Z*** are the design matrices of ***b*** (including sex and batch), and ***u***, respectively. It was assumed that ***u*** and ***e*** follow multivariate normal distributions, ***u*** ~ *N*(**0**, ) and ***e*** ~ *N*(**0**, ), respectively, where is the additive genetic variance and is the residual variance. ***A*** is the genomic relationship matrix and it is constructed using the genotyped SNPs according to VanRaden’s method [40]. *g* is the substitution effect of current focus SNP, and ***m*** is its index vector. With the large number of SNPs and extensive linkage disequilibrium (LD), Bonferroni correction is overly stringent for significance testing. Instead, threshold *P* values of suggestive and genome-wide significance were set as 1 × 10^–6^ and 5 × 10^–8^, respectively, following [41, 42]. To visualize the GWAS results, Manhattan and Quantile–quantile (QQ) plots were produced using R (version 4.3.0).

The PVE (%) of each genome-wide significant SNP was calculated using its estimated allelic effect *β* and standard error *se*(*β*), and its MAF, following [43]:

2 $$ PVE = \frac{{2\beta^{2} \times MAF \times (1{-}MAF)}}{{2\beta^{2} \times MAF \times (1{-}MAF) + 2N \times MAF \times (1{-}MAF) \times se(\beta )^{2} }}, $$

where *N* is the sample size.

After GWAS, LD among SNPs and gene annotation in the regions of genomic loci were displayed with LocusZoom [44] using *Sscrofa*11.1 as the reference genome.

SNP-based heritability of BW and BMI was estimated using GCTA-REML [45] under the LMM corresponding to Eq. (1) without the SNP effect. Sex and batch were fitted as fixed covariates, and the random additive genetic effect was modeled using a genomic relationship matrix constructed from the qualified SNPs.

### Fine mapping

To refine the GWAS association signals, fine-mapping was performed using the Sum of Single Effects (SuSiE) model implemented in the susieR package (v0.12.41) [32]. SuSiE analyses integrated GWAS summary statistics with a population-matched LD reference panel that was derived from whole-genome sequences of the Shanxia Long Black pig population, allowing joint modeling of multiple causal variants per locus. For each locus, the maximum number of causal signals (*L*) was set to 10, which is a conservative value that accommodates moderate allelic heterogeneity and aligns with standard practices in fine-mapping of complex traits [32]. Posterior inclusion probabilities (PIP) were computed for all variants, and 95% credible sets were constructed to identify those most likely to be causal. The variants with PIP > 0.1 were proposed as candidate variations for further analysis [46].

Fine-mapping was restricted to variants within a ± 500 kb window centered on each lead SNP, because the pairwise LD (*r*^2^) dropped rapidly with distance and reached the baseline within ~ 100–200 kb in Shanxia Long Black pig (Additional file 3: Fig. S1). Therefore, the ± 500 kb interval almost captured all variants in high LD with the lead SNP and minimized the work to exclude the weak LD variants.

### Chromatin state segmentation and visualization

To identify tissue-enriched regulatory elements of BW and BMI, we integrated fine-mapped intervals with chromatin state maps for 14 porcine tissues, covering 15 states, including the islands of promoter (TssA, TssAHet, TssBiv), transcribed (TxFlnk, TxFlnkWk, TxFlnkHet), enhancer (EnhA, EnhAMe, EnhAWk, EnhAHet, EnhPois), repressive (Repr, ReprWk), quiescent (Qui), and ATAC [47].

As genetic variants can act at potentially large distances, we next sought to identify the genes that are targeted by the regulatory region associated with BW and BMI, whose expression may be affected by the identified candidate functional variants.

### Hi-C data processing and visualization

To predict putative target genes, Hi-C sequencing was performed on subcutaneous adipose tissue (backfat) from a single pig from a previously described population [48]. In situ Hi-C libraries were generated following the protocol of Rao et al. [49], with the inclusion of a custom MboI restriction enzyme recognition site. Raw Hi-C reads were first subjected to quality assessment using FastQC and reads shorter than 100 bp were excluded. The clean data were then processed using the Juicer pipeline with default parameters. Contact maps were generated at 5 kb resolutions, which passed the Juicer resolution prediction [50].

We additionally collected 5-kb-resolution.hic files for brain and liver tissues and performed the following analyses [51]. Topologically associating domains (TADs) were identified at 5 kb resolution using the HiCExplorer algorithm (v3.5.1) [52], while chromatin loops were detected at 5 kb resolution using Mustache (v1.0.1) [53]. Finally, the genomic tracks were visualized using the pyGenomeTracks [54].

### Gene-based association study

To investigate the association of genes with BW and BMI, genome-wide gene association analyses were performed using MAGMA v1.10b [55]. A total of 32,130 genes were annotated from a high-quality reference genome assembled in our laboratory [37], and 29,452 genes were qualified and used for the gene association analysis. The significance threshold *P* value was set at 1.70 × 10^–6^ based on 0.05 divided by the number of qualified genes. We applied MAGMA to test genome-wide gene-level associations, providing an annotation-agnostic approach to detect aggregate effects that may escape single-SNP analyses.

### *Basenji* convolutional neural network (CNN) training and variant effect prediction

#### Data processing

Epigenomic datasets, including transposase-accessible chromatin sequencing (ATAC-seq) and chromatin immunoprecipitation sequencing (ChIP-seq) from adipose, brain, and liver tissues, were downloaded from previous publications [56, 57] and utilized as prediction references [33]. We adapted the *Basenji* CNN framework to the pig genome (*Sus scrofa* 11.1) to predict the regulatory effects of non-coding variants. This model takes 1,344 bp genomic DNA sequences represented as one-hot encoded matrices (A, C, G, and T channels) as input and outputs quantitative signals for chromatin accessibility and histone modifications, using a gap-annotated Ensembl FASTA reference genome.

Data preprocessing and dataset construction were performed using basenji_data.py [58] with the following parameters: --local --p 20 --r 4096 --w 192 --l 1344 --v 0.12 --t 0.12 --stride 192 --stride_test 192 --crop 576. This fixed the input sequence length at 1344 bp, processed data at a resolution of 4096 bp (with predictions aggregated across bins), applied a stride of 192 bp for sequence sampling, and cropped outputs by 576 bp to focus predictions on the central region. To prevent information leakage from local LD or sequence similarity, chromosomes were split into training (76%), validation (12%) and test (12%) regions for all tissue-specific models.

#### Model training

CNN models were trained using the basenji_train.py [59] on an NVIDIA A100 GPU. The architecture consisted of an initial convolutional layer (288 filters, kernel size 17 bp, pooling factor 3), followed by a six-block convolutional tower with progressively increasing filters (from 288 to 512; kernel size 5 bp, pooling factor 2 per block), an additional convolutional layer (256 filters, kernel size 1 bp), and a fully connected layer (768 units, dropout rate 0.2). The output layer comprised 11 sigmoid-activated units, one for each epigenomic track. Training employed stochastic gradient descent (batch size 64, initial learning rate 0.005, and momentum 0.98) with binary cross-entropy loss. Early stopping was implemented if validation loss did not improve for 12 consecutive epochs.

#### Model evaluation

The predictive performance of *Basenji* models, trained independently on adipose, brain, and liver epigenomic data, was evaluated solely on the reserved test set utilizing basenji_test.py. Prediction accuracy was measured by the area under the receiver operating characteristic curve (AUROC) and the area under the precision-recall curve (AUPRC), providing an unbiased evaluation of model generalization.

#### SNP activity difference score prediction and mutagenesis

SNP Activity Difference (SAD) scores were predicted using basenji_sad.py to evaluate allelic effects on chromatin accessibility and histone modification at candidate loci. Variants with high SAD scores were prioritized as functional candidates. In silico saturation mutagenesis was conducted using basenji_sat_vcf.py, testing all four nucleotides at each position. The loss score represents the maximum decrease in chromatin-state signal when mutating to a non-reference nucleotide, and the gain score represents the maximum increase in field [59].

### Genomic prediction

To assess the predictive utility of prioritized candidate variants for BW and BMI, we performed genomic prediction for BW and BMI using Shanxia Long Black pigs and simulated pig breeding populations with three marker densities (60 K, 140 K, and 660 K). The simulated genomes comprised 18 autosomes, with chromosome lengths defined according to the Ensembl *Sus scrofa* (v11.1) reference genome. Candidate variants prioritized from respective GWAS were incorporated as fixed effects into the two statistical models, from which four distinct breeding value estimators (FM_PBV, MM_PBV, Genomic estimated breeding values (GEBV), and QTL-fixed GEBV) were used to evaluate prediction performance.

#### Simulation population

To evaluate the accuracy of the genomic prediction methods, we performed simulations in QMSim 2.0 [60]. Phenotypes were simulated with heritability 0.4, phenotypic variance 1.0, and a QTL heritability of 0.2. SNP panels comprising 60 K, 140 K, or 660 K markers were distributed across 18 chromosomes (61–274 cM), with markers and QTLs positioned randomly.

To mimic the polygenic architecture of pig growth traits, gene action was assumed to be additive, and QTL effects were sampled from a gamma distribution with a shape parameter of 0.4, generating many small-effect QTLs and a few large-effect QTLs. Initial allele frequencies for both markers and QTLs were set to 0.5. A historical population of 5000 individuals was randomly mated for 1000 generations without selection to generate allele-frequency drift and LD structure.

After the historical phase, a recent population was founded with 20 boars and 600 sows, corresponding to a mating ratio of 1:30. Litter sizes of 12, 15, 17, and 21 were assigned probabilities of 0.24, 0.35, 0.20, and 0.21, respectively, with an expected male progeny proportion of 0.5. The recent population was simulated for seven generations under positive assortative mating based on true breeding values, with replacement rates of 80% for sires and 20% for dams. Low-ranking animals were culled based on true breeding values, and estimated breeding values (EBVs) were obtained using pedigree-based best linear unbiased prediction (BLUP).

Genotypic and phenotypic data of 9636, 9665, and 9597 individuals with 60, 140, and 660 K SNPs from the seventh generation of the recent population were analyzed using the parseQMSim function in wildsim (R 4.3.0) (available at https://github.com/susjoh/wildsim) and quality-controlled with PLINK (1.90b7) [35]. SNPs and individuals were retained if they met MAF > 0.01, HWE *P* > 1 × 10^–6^, and call rate > 0.95. After filtering, 43,256, 112,903, and 552,830 SNPs remained for the three panels, respectively. The qualified datasets were performed GWAS using the model described in Eq. (1) to identify putative QTLs and conducted genomic prediction to assess predictive performance.

#### BLUP and GBLUP models

For BW/BMI traits and simulated datasets, candidate functional variants identified from GWAS were explicitly incorporated as fixed effects in genomic prediction models. Two statistical models were fitted: a fixed-effect model and a mixed linear model.

The fixed-effect model was formulated as:

3 * y = Xb + mg + e, *

where ***y*** is the vector of phenotypic observations, ***b*** and ***X*** represent the vector of fix effects and its incidence matrix, ***m*** is the dosage vector for the candidate variant coded as 0, 1 or 2 copies of the alterative allele effect and fitted as fixed-effect covariates, *g* is the additive allelic effect of the candidate variant, and ***e*** is the vector of residual errors.

The fixed-effect model was extended to the mixed linear model by adding a random polygenic term:

4 * y = Xb + mg + Zu + e, *

where ***u*** is the vector of genome-wide random polygenic effects, assumed to follow ***u*** ~ *N*(**0**, $A\sigma_u^2$), where ***A*** is the genomic relationship matrix constructed from genome-wide SNPs and $\sigma_u^2$ is the additive effect variance. ***Z*** is the incidence matrix of ***u***.

The fixed-effect model in Eq. (3) was fitted in R (version 4.3.0), whereas the mixed linear model described in Eq. (4) was fitted using HIBLUP [61].

#### Estimation of breeding values

To dissect the specific contributions of candidate functional variants and polygenic background, we derived four distinct breeding value estimators based on the fitted models:

1. *FM_PBV*: Predicted breeding values are based on the fixed effects of candidate functional variants (***a*** = ***m****g*), estimated using the fixed-effect model.
2. *MM_PBV*: Similar to FM_PBV (***a*** = ***m****g*), but the fixed effects are estimated using the linear mixed model.
3. *GEBV*: Genomic estimated breeding values are equal to genome-wide random polygenic effects (***a*** = ***u***), corresponding to GBLUP [21, 40].
4. *QTL-fixed GEBV*: Composite breeding values are the sum of candidate functional variants and the polygenic effects (***a*** = ***m****g* + ***u***), estimated using HIBLUP [61].

#### Cross-validation and predictive performance

To obtain a robust and unbiased assessment of genomic prediction performance, a tenfold cross-validation strategy was implemented in this study. Shanxia Long Black pig and simulated datasets were randomly partitioned into 10 non-overlapping subsets, respectively. Each fold was in turn as test data, and GWAS was performed de novo to identify the significant SNPs using the other nine training subsets. Then, these identified SNPs were used to construct the prediction model in the training data, and the GEBVs of the test data were predicted. To minimize sampling bias, the entire process was independently replicated five times. Prediction performance was evaluated via 50 cross-validation replicates (10 folds × 5 runs) [62, 63]. For Shanxia Long Black pig data, prediction accuracy was measured as the Pearson correlation coefficient between GEBVs and observed phenotypes. For the simulated data, the prediction accuracy was the Pearson correlation coefficient between GEBVs and the true breeding values (TBVs).

## Results

### Summary of phenotypes

Table 1 showed the BW and BMI descriptive statistics of Shanxia Black pigs. Coefficients of variations ranged from 10.4 to 13.9%. Phenotypic distributions of the traits are shown in Additional file 3: Fig. S2. The SNP-based heritability estimate was 0.23 ± 0.05 for BW and 0.37 ± 0.05 for BMI.

**Table 1:** Descriptive statistics of phenotypes

Trait	Number	Mean ± SD	CV, %	*h*^2^ ± SE
Body weight, kg	1226	107.32 ± 14.90	13.88	0.23 ± 0.05
Body mass index, kg/m^2^	1226	85.99 ± 8.96	10.42	0.37 ± 0.05

SD and CV are the acronyms of standard deviation and coefficient of variation, respectively

### Genome-wide significant QTL for BW and BMI

**Fig. 1:**
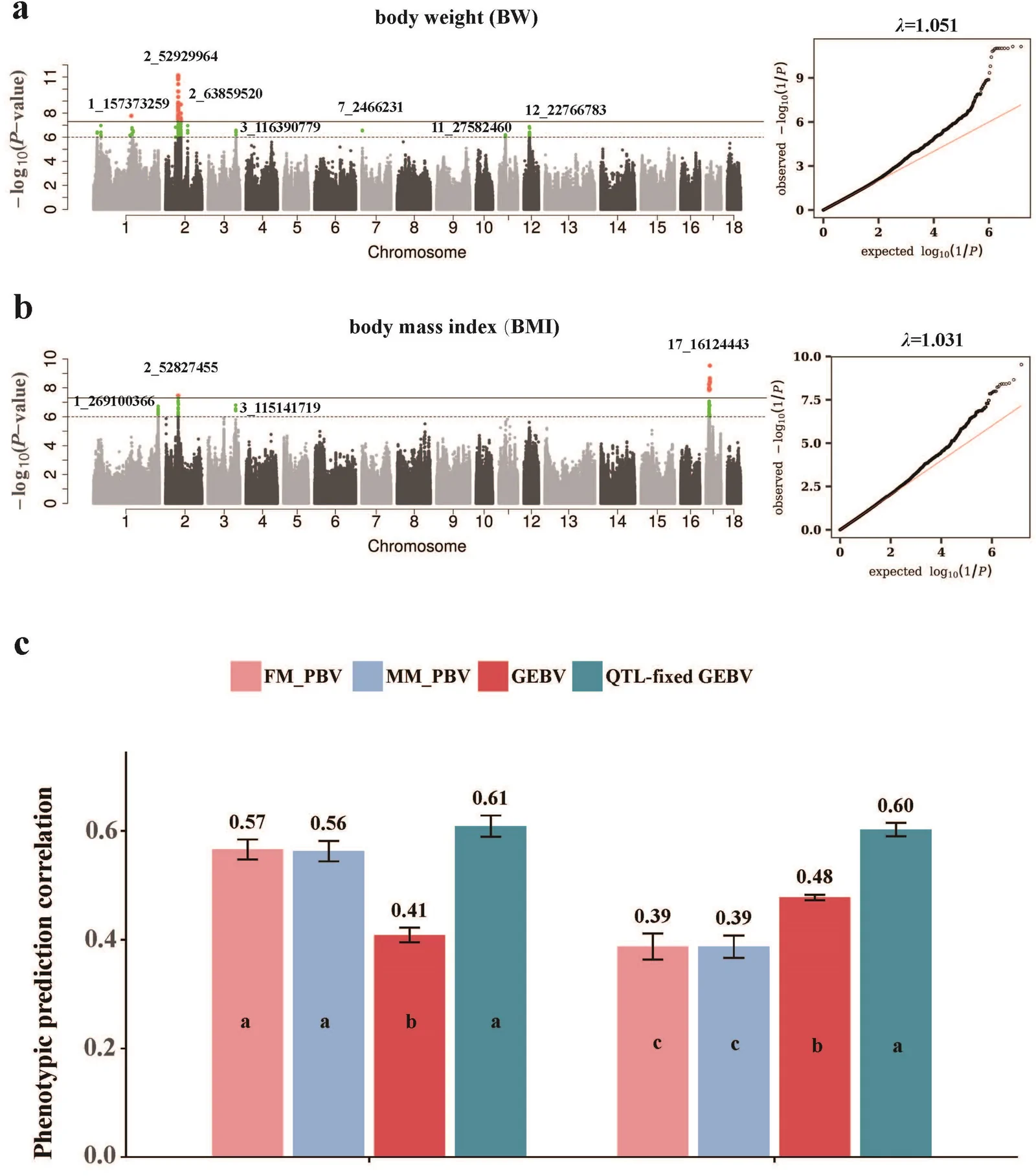
Results of GWAS and genomic prediction for BW and BMI. **a** and **b** are the Manhattan and QQ plots for BW and BMI, respectively. Horizontal lines are the genome-wide (*P* = 5 × 10^–8^, solid) and suggestive (*P* = 1 × 10^–6^, dotted) significance thresholds. Red lines in QQ plots are the expected distribution under the null hypothesis. The genomic inflation factor (*λ*) is indicated for each trait. **c** Comparing the predictive performance of the estimated breeding value with four methods in the Shanxia Long Black pig population. FM_PBV: estimated breeding values (***a*** = ***m****g*) from a fixed-effect model with only the candidate functional variants fitted as fixed effects; MM_PBV: estimated breeding values (***a*** = ***m****g*) from a mixed linear model with candidate functional variants as fixed effects and genome-wide polygenic effects as random effects; GEBV: genomic estimated breeding values (***a*** = ***u***) obtained from a GBLUP model with genome-wide random polygenic effects; QTL-fixed GEBV: predicted breeding values (***a*** = ***m****g* + ***u***) obtained from a mixed linear model with candidate functional variants as fixed effects and genome-wide polygenic effects as random effects. Different lowercase letters (a, b, c) indicate significant differences among the four estimated breeding value methods within each trait-marker density combination (one-way ANOVA with Tukey’s HSD test, *P* < 0.05)

Eight genomic regions reached genome-wide significance for BW, with lead SNPs mapped to SSC1 (157.37 Mb), SSC2 (52.93, 63.86, and 64.30 Mb), SSC3 (116.39 Mb), SSC7 (2.47 Mb), SSC11 (27.58 Mb), and SSC12 (22.77 Mb) (Fig. 1a; Table 2). For BMI, four significant regions were detected on SSC1 (269.10 Mb), SSC2 (52.83 Mb), SSC3 (115.14 Mb), and SSC17 (16.12 Mb) (Fig. 1b; Table 2), respectively. QQ plots indicated minimal population stratification (*λ* = 1.051 for BW and *λ* = 1.031 for BMI) (Fig. 1a–b).

**Table 2:** The lead SNP of each genomic region identified by GWAS

Trait	Marker	Chr	Position, Mb	Alleles	MAF	Effect ± SE	*P-*value	PVE, %
BW	1_157373259	1	157.37	A/T	0.316	− 3.89 ± 0.68	1.69E-08	2.65
	2_52929964	2	52.93	T/C	0.351	4.31 ± 0.62	7.28E-12	3.87
	2_63859520	2	63.86	A/G	0.036	− 9.19 ± 1.52	1.92E-09	3.00
	2_64304180	2	64.30	A/G	0.033	− 8.77 ± 1.59	3.93E-08	2.50
	3_116390779	3	116.39	C/G	0.424	− 3.23 ± 0.62	2.63E-07	2.20
	7_2466231	7	2.47	A/G	0.090	5.23 ± 1.01	2.63E-07	2.18
	11_27582460	11	27.58	C/A	0.243	− 3.62 ± 0.72	6.34E-07	2.06
	12_22766783	12	22.77	G/A	0.049	− 7.60 ± 1.43	1.34E-07	2.30
BMI	1_269100366	1	269.10	T/C	0.281	2.19 ± 0.42	1.88E-07	2.22
	2_52827455	2	52.83	C/T	0.409	2.01 ± 0.36	3.45E-08	2.48
	3_115141719	3	115.14	G/A	0.204	2.53 ± 0.48	1.57E-07	2.24
	17_16124443	17	16.12	G/A	0.475	− 2.72 ± 0.43	2.92E-10	3.33

Chr, MAF, and PVE are the abbreviations for chromosome, minor allele frequency, and the proportion of genetic variance explained, respectively

Notably, GWAS signals for BW and BMI converged on a shared region at about 52.8 Mb on SSC2, encompassing a cluster of olfactory receptor genes, including *OR2T27*, *OR2T6*, and *OR2T1* (Additional file 3: Fig. S3a and g).

### SNP prioritization based on LD and fine-mapping

**Fig. 2:**
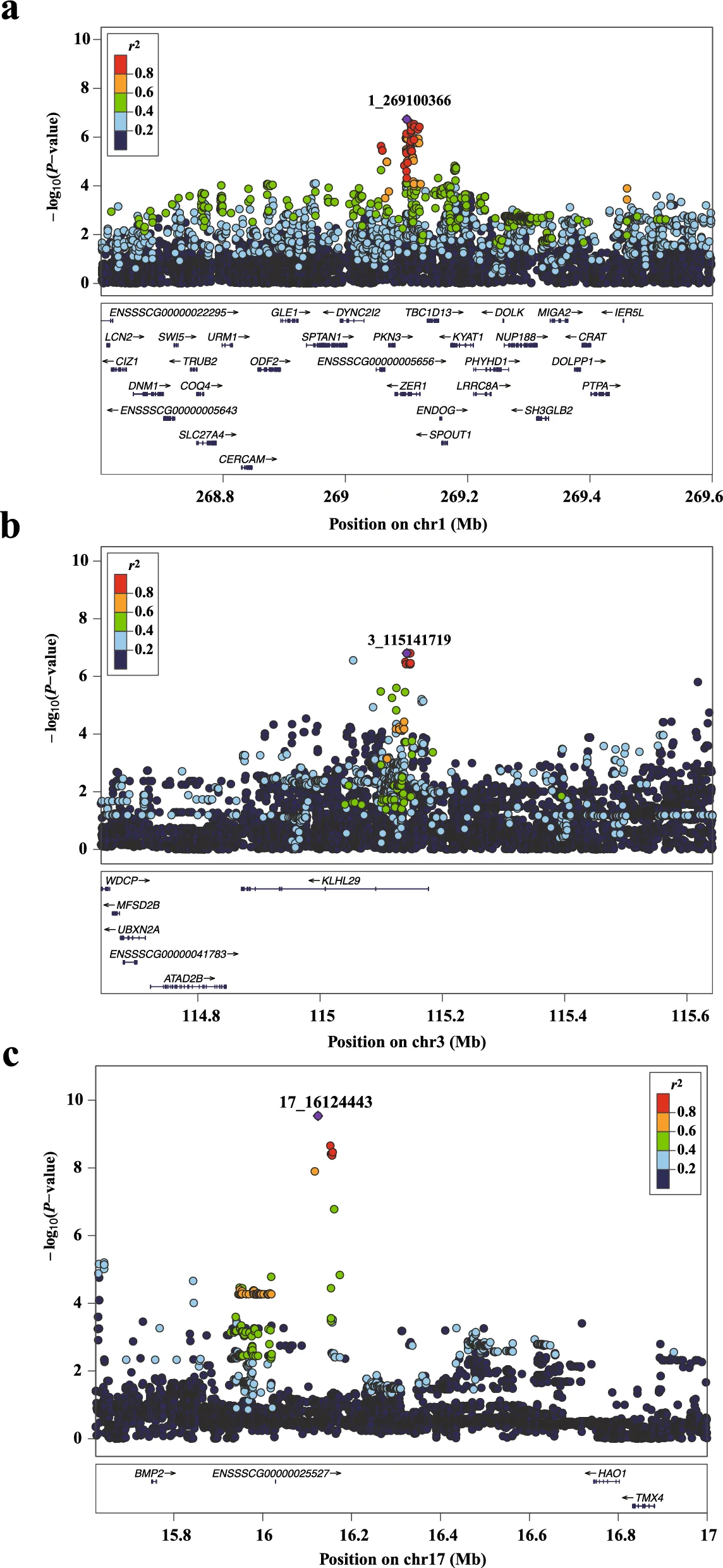
Genomic regions associated with BMI on SSC1, SSC3 and SSC17. **a** from 268,600,366 to 269,600,366 on SSC1. **b** from 114,641,719 to 115,641,719 on SSC3. **c** from 15,624,443 to 17,000,000 on SSC17. In each plot, the top panel shows the negative logarithms (base 10) of *P* values and linkage disequilibriumd egrees (*r*^2^) to the top SNP, and the bottom panel shows the annotated genes in the region

To fine-map QTLs, LD between lead SNPs and surrounding variants were assessed within a 500-kb window. Variants with *r*^2^ > 0.8 were defined as belonging to the local LD block. For BW, six distinct LD blocks were identified (Table 3), with sizes ranging from 18.85 to 643.07 kb. These were distributed across SSC2 (two blocks), SSC3, SSC7, SSC11, and SSC12 (Additional file 3: Fig. S3a–f). For BMI, four LD blocks were detected, located on SSC1 (62.82 kb), SSC2 (57.99 kb), SSC3 (8.43 kb), and SSC17 (33.12 kb) (Fig. 2a–c; Additional file 3: Fig. S3g).

**Table 3:** Significant genomic regions of body size traits identified using GWAS

Traits	Lead SNP	Chromosome	Candidate QTL, bp	Size of LD Block, kb
Body weight	2_52929964	2	52,838,612–52,943,942	105.33
	2_63859520	2	63,811,912–64,339,157	527.25
	3_116390779	3	116,296,662–116,441,118	144.46
	7_2466231	7	2,014,550–2,657,615	643.07
	11_27582460	11	27,580,050–27,598,895	18.85
	12_22766783	12	22,724,702–23,255,707	531.01
Body mass index	1_269100366	1	269,058,230–269,121,052	62.82
	2_52827455	2	52,827,455–52,885,433	57.99
	3_115141719	3	115,139,440–115,147,865	8.43
	17_16124443	17	16,124,443–16,157,566	33.12

Within these LD blocks, fine-mapping was conducted using SuSiE [32] to identify candidate functional variants for BW and BMI. Variants with PIPs > 0.1 and the lead SNPs were considered candidate variants, resulting in 29 candidate functional variants for BW (1 on SSC1, 14 on SSC2, 9 on SSC3, 2 on SSC7, 2 on SSC11, 1 on SSC12) and 12 for BMI (1 on SSC1, 4 on SSC2, 1 on SSC3, 6 on SSC17) (Additional file 1: Table S1).

### Multi-omics prioritization of regulatory variants and candidate genes selection

**Fig. 3:**
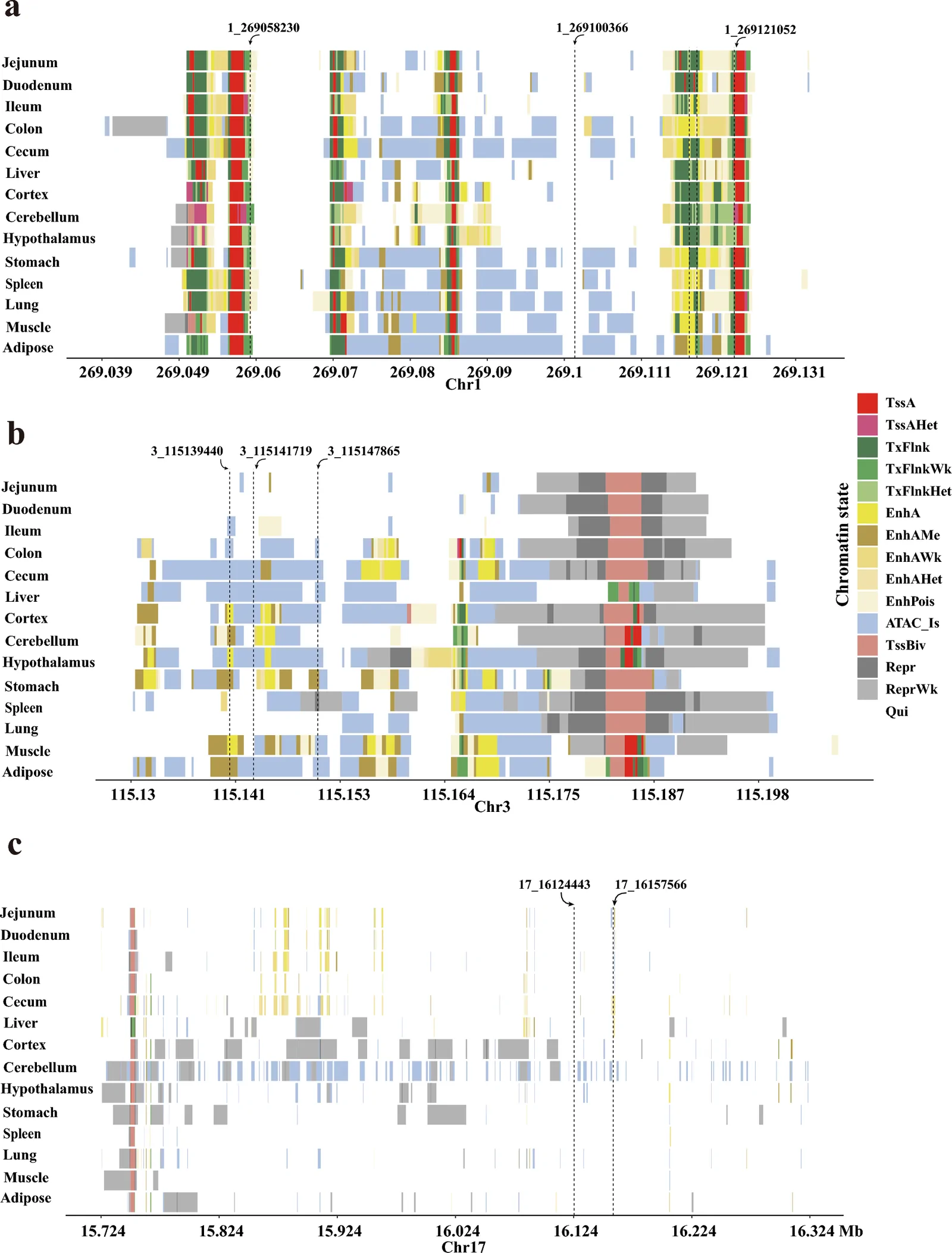
Chromatin state annotation of quantitative trait loci for BMI. **a**–**c** Chromatin state landscapes of 14 porcine tissues at the loci of BMI on SSC1, SSC3 and SSC17, respectively. The tracks depict 15 distinct chromatin states, categorizing functional elements into active promoters (TssA, TssAHet, and TssBiv), TSS-proximal transcribed regions (TxFlnk, TxFlnkWk, and TxFlnkHet), active and poised enhancers (EnhA, EnhAMe, EnhAWk, EnhAHet, and EnhPois), repressed regions (Repr, and ReprWk), and quiescent chromatin (Qui). Accessible chromatin peaks are marked as ATAC islands

To pinpoint tissues where candidate functional variants exert effects, we integrated chromatin state annotations (ATAC-seq and ChIP-seq) across 14 porcine tissues with BW- and BMI-associated QTLs. The BW QTL on SSC2 (52,838,612–52,943,942 bp) overlapped a cerebellar open chromatin region (Additional file 3: Figure S4a). For BMI, the SSC1 QTL (269,058,230–269,121,052 bp) overlapped with enhancer in adipose tissue (Fig. 3a), the SSC3 QTL (115,139,440–115,147,865 bp) encompassed brain-active enhancers (Fig. 3b), and the SSC17 QTL(16,124,443–16,157,566 bp) coincided with a weak liver enhancer (Fig. 3c).

**Fig. 4:**
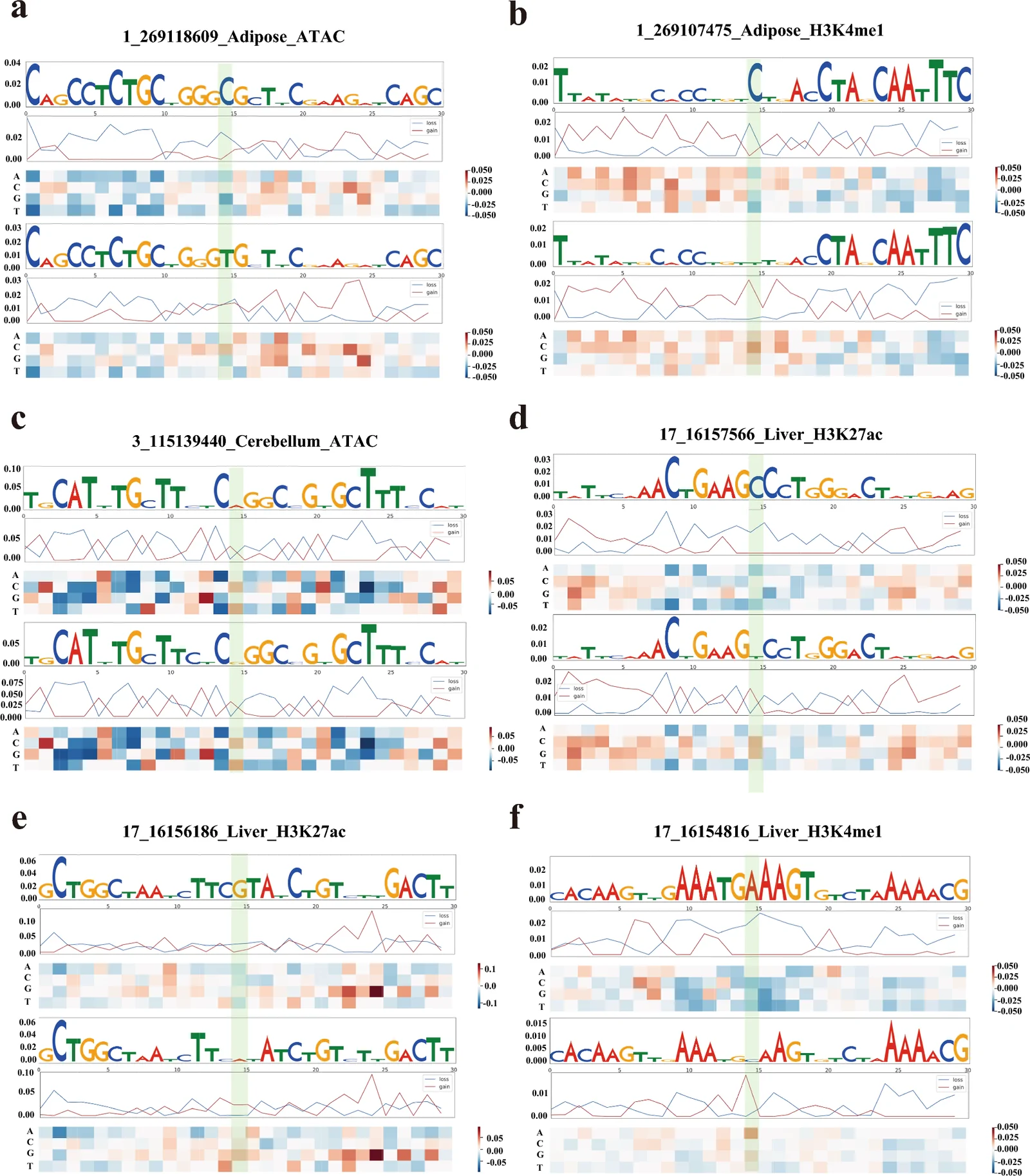
Predicting the allele-dependent effects of candidate variants on chromatin activity via In silico saturation mutagenesis. **a**–**b** rs321875264 (SSC1_269118609) and rs329346269 (SSC1_269107475) in adipose tissue, respectively. **c** rs329388918 (SSC3_115139440) in the cerebellum. **d**–**f**, rs330068434 (SSC17_16157566), rs345777289 (SSC17_16156186), and rs337262650 (SSC17_16154816) in the liver, respectively. ATAC-seq, H3K4me1, and H3K27ac mark chromatin accessibility and enhancer-associated histone modifications, respectively. Loss score represents themaximum decrease in predicted chromatin-state signal upon substitution with a non-reference nucleotide, whereas gain score represents maximum increase in signal. Each x-axis position denotes a nucleotide, and heatmap values represent predicted effects of substituting that position with A, C, G, or T

We next assessed regulatory variants using a *Basenji* CNN [33] trained on epigenetic profiles from adipose, brain, and liver tissues to predict SNP effects (*P* < 1.0 × 10^–6^) within fine-mapped QTLs. The model showed good performance (AUROC: 0.886, 0.863, 0.896; AUPRC: 0.593, 0.562, 0.558 for adipose, liver, and brain, respectively; Additional file 3: Figure S5a–b). In silico mutagenesis of 30-bp flanking sequences quantified allele-specific chromatin effect using SAD scores. For BW, the *C* allele at SSC2_52929964 (*C* vs. *T*) enhanced cerebellar accessibility (SAD = 0.03; Additional file 3: Figure S4c). For BMI, tissue effects included adipose—increased accessibility (*C* vs. *T* at rs321875264, SSC1_269118609; SAD = 0.02; Fig. 4a) and H3K4me1 (*C* vs. *T* at rs329346269, SSC1_269107475; SAD = 0.015; Fig. 4b); brain—elevated cerebellar accessibility (*A* vs. *G* at rs329388918, SSC3_115139440; SAD = 0.02; Fig. 4c); and liver—boosted H3K27ac (*C* vs. *T* at rs330068434, SSC17_16157566; SAD = 0.01; Fig. 4d; *G* vs. *A* at rs345777289, SSC17_16156186; SAD = 0.02; Fig. 4e) and H3K4me1 (*A* vs. *C* at rs337262650, SSC17_16154816; SAD = 0.01; Fig. 4f).

**Fig. 5:**
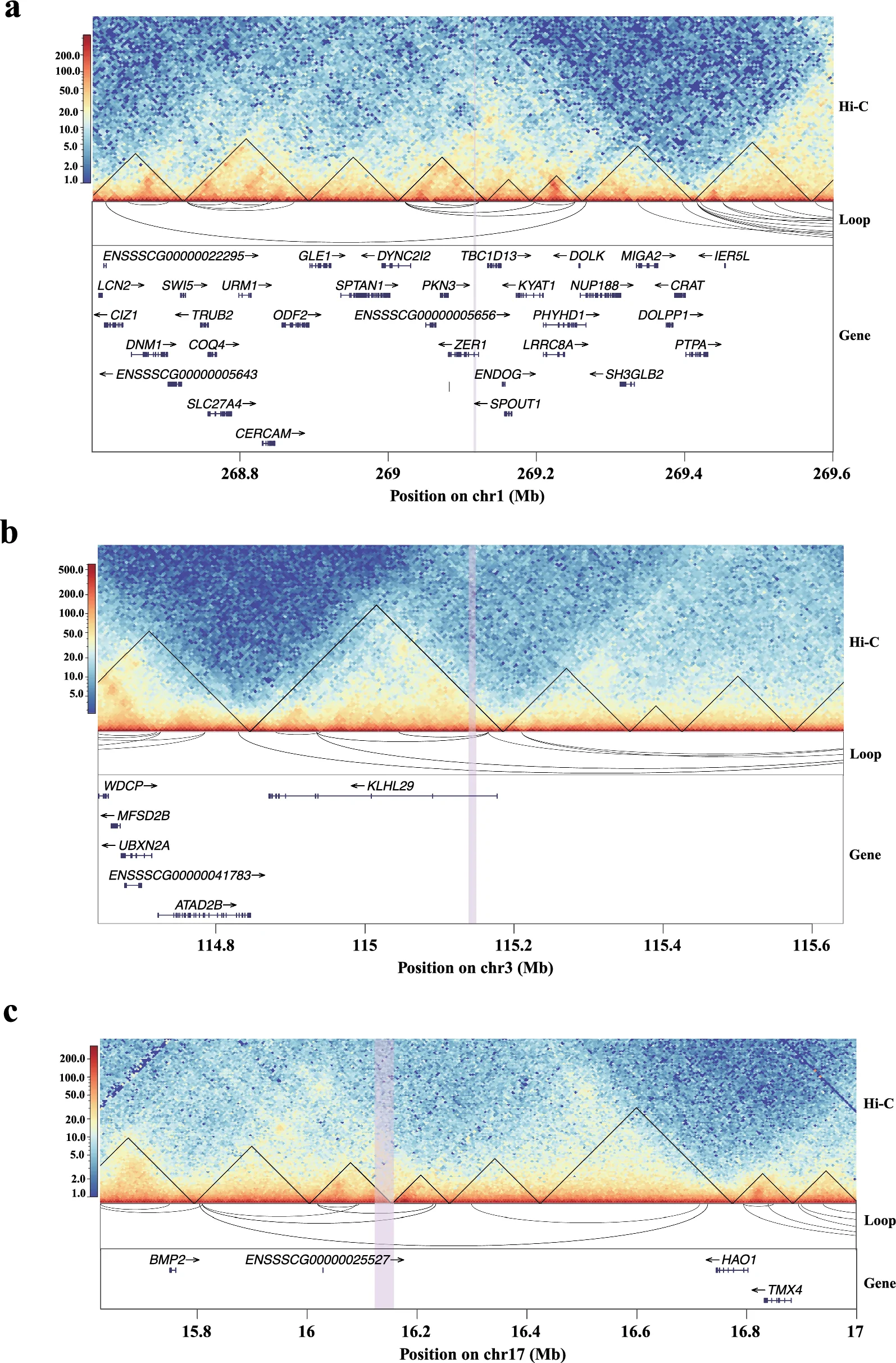
Chromatin interactions and regulatory architecture of quantitative trait loci for BMI. **a** SSC1 (adipose); **b** SSC3 (brain); **c** SSC17 (liver). Top: Hi-C contact matrices at 5-kb resolution with TAD boundaries delineated. Middle: Significant chromatin loops identified across the same intervals. Bottom: Gene models and annotations within the corresponding regions

To nominate target genes, we leveraged Hi-C data (5-kb resolution) from adipose, brain, and liver to delineate chromatin loops and TADs. In adipose tissue, the BMI-associated SSC1 QTL harbored an enhancer (variant SSC1_269100366) within a TAD spanning *DYNC2I2*, *PKN3*, *ENSSSCG00000005656*, and *ZER1*. Hi-C data revealed chromatin loops connecting this TAD to an adjacent TAD containing *KYAT1*, *PHYHD1*, *LRRC8A*, *DOLK*, and *NUP188* (Fig. 5a). The lead SNP was located within *ZER1*, nominating *ZER1* as the likely candidate gene underlying this BMI QTL, corroborated by MAGMA [55] (*P* = 8.00 × 10^–7^, Additional file 2: Table S2). In the brain, the BMI QTL on SSC3 (variant SSC3_115141719) overlapped a brain enhancer in the *KLHL29* TAD, nominating *KLHL29* (Fig. 5b). The BMI-associated SSC17 QTL (variant SSC17_16124443) connected to *ENSSSCG00000025527* and *HAO1* in the liver via bridging chromatin loops across three nearby TADs (Fig. 5c). In the liver and kidney, *HAO1* is highly expressed, especially in hepatocytes and proximal tubule cells (PigGTEx; Additional file 3: Fig. S6a–b) [64]. In general, these multi-omics analyses revealed the tissue-specific regulatory architecture underlying porcine growth.

### Predictive accuracy of GP

In Shanxia Long Black pigs, because the true breeding values were unavailable, the predictive accuracy was measured as the Pearson correlation between predicted values and observed phenotypes. For BW, QTL-fixed GEBV (*r* = 0.61 ± 0.02), FM_PBV (*r* = 0.57 ± 0.02) and MM_PBV (*r* = 0.56 ± 0.01) were significantly more accurate than GEBV (*r* = 0.41 ± 0.02). For BMI, QTL-fixed GEBV (*r* = 0.60 ± 0.01) was significantly more accurate than GEBV (*r* = 0.48 ± 0.01), and the latter was significantly higher than those of FM_PBV (*r* = 0.39 ± 0.02) and MM_PBV (*r* = 0.39 ± 0.02) (Fig. 1c). For both BW and BMI, QTL-fixed GEBV was the most accurate method, and its predictive accuracy was improved with 7.01–53.85% comparing to the other 3 methods.

**Fig. 6:**
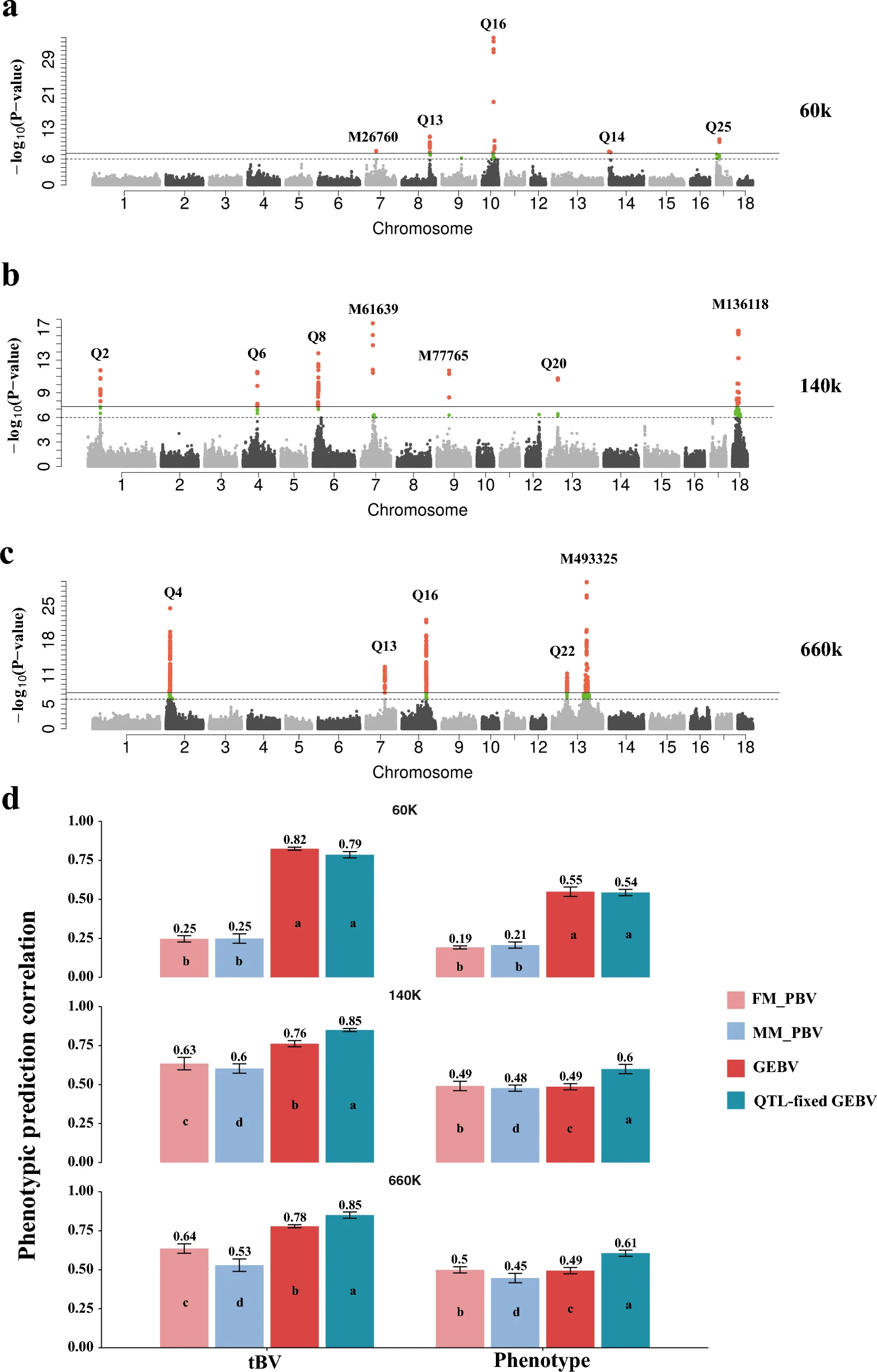
GWAS performance and genomic prediction accuracy in the simulated data. **a**–**c** Manhattan plots of the simulated data with 60K, 140K, and 660K SNPs, respectively. Horizontal lines indicate genome-wide (*P* = 5 × 10^–8^, solid) and suggestive (*P* = 1 × 10^–8^, dashed) significance thresholds. **d** Comparing the predictive performance using different marker densities. FM_PBV: estimated breeding values (***a*** = ***m****g*) from a fixed-effect model with only the candidate functional variants fitted as fixed effects; MM_PBV: estimated breeding values (***a*** = ***m****g*)from a mixed linear model with candidate functional variants as fixed effects and genome-wide polygenic effects as random effects; GEBV: genomic estimated breeding values (***a*** = ***u***) obtained from a GBLUP model with genome-wide random polygenic effects; QTL-fixed GEBV: predicted breeding values (***a*** = ***m****g* + ***u***)obtained from a mixed linear model with candidate functional variants as fixed effects and genome-wide polygenic effects as random effects; tBV, true breeding values. Different lowercase letters (a, b, c, d) indicate significant differences among the four estimated breeding value methods within each trait-marker density combination (one-way ANOVA with Tukey’s HSD test, *P* < 0.05)

In the simulated data, the QTLs (*P* < 5 × 10^–8^; Fig. 6a–c) were detected with GWAS, and then they were fitted in the genomic prediction. Because the true breeding values (tBV) were available in the simulated data, the predictive accuracy was measured as the Pearson correlation between the predicted values and tBV. At 60K marker density, GEBV (*r* = 0.82 ± 0.01) and QTL-fixed GEBV (*r* = 0.79 ± 0.01) significantly outperformed than FM_PBV (*r* = 0.25 ± 0.03) and MM_PBV (*r* = 0.25 ± 0.02). At higher marker densities (140K and 660K), QTL-fixed GEBV (*r* = 0.85 ± 0.01 and *r* = 0.85 ± 0.01) significantly outperformed than GEBV (*r* = 0.76 ± 0.01 and *r* = 0.78 ± 0.01), and GEBV performed significantly better than FM_PBV (*r* = 0.63 ± 0.01 and *r* = 0.64 ± 0.01). MM_PBV (*r* = 0.60 ± 0.01 and *r* = 0.53 ± 0.01) was the worst method among the 4 method (Tukey-adjusted pairwise comparisons, *P* < 0.05; Fig. 6d). The Pearson correlations of the predicted values with phenotypes were as same as those with tBV, but the correlations were weaker.

In conclusion, incorporating significant variants as fixed effects enhanced prediction accuracy across diverse scenarios, especially with high marker density. Prior biological information could significantly improve the performance of genomic prediction.

## Discussion

Recent studies have demonstrated extensive pleiotropy among loci, SNPs, and gene sets associated with complex polygenic traits in pigs [2, 7–10, 65–67]. Identifying high-confidence candidate functional variants remains difficult because most GWAS signals map to non-coding regions with poor mapping resolution. In this study, we addressed this challenge by delineating the multi-tissue regulatory architecture of BW and BMI in Shanxia Long Black pigs. By integrating fine-mapping with multi-omics layers—specifically chromatin state annotations, Hi-C, and deep-learning-based *Basenji* predictions—we prioritized candidate variants and linked them to their effector tissues and candidate target genes. These findings provide mechanistic insights into body size regulation and enhance the genomic prediction accuracy of BW and BMI.

### Genetic architecture of BW and BMI

We discovered 10 QTLs, including 8 for BW and 4 for BMI, together with 45 plausible candidate SNPs (29 associated to BW and 15 to BMI). These QTLs account for just a small proportion of genetic variances for BW (20.76%) and BMI (10.27%), indicating the polygenic nature of those traits. Hi-C analysis in brain tissue localized this signal within a single TAD. Brain regulatory elements enrich on SSC2, and an olfactory receptor (*OR*) cluster locates at the QTL regions. The olfactory receptor genes involve sensory-neural pathways and modulate feeding behavior, and feed intake is a potential key intermediate linking this locus to BW and BMI. *OR2T27* was the sole olfactory receptor gene annotated within this TAD boundary, effectively prioritizing it over neighboring paralogs (Additional file 3: Fig. S4b). These findings highlight a shared regulatory architecture and position *OR2T27* as a putative driver of growth variation, potentially through neural-mediated pathways. Other BMI loci showed enhancer activity in adipose, brain, and liver tissues, indicating a multi-tissue regulatory basis. Integrative Hi-C and *Basenji* CNN analyses linked some fine-mapped variants to tissue-specific target genes, such as *ZER1* (rs321875264, rs329346269), *KLHL29* (rs329388918), and *HAO1* (rs330068434, rs345777289, rs337262650), and revealed diverse regulatory mechanisms in these polygenic traits.

### Functional insights into candidate effector genes

By integrating genetic, epigenetic, and transcriptomic evidence, *ZER1*, *KLHL29*, and *HAO1* were considered as high-confidence effector genes, while *OR2T2*7 was a putative candidate gene. *ZER1* encodes a substrate adaptor for the Cullin-2 E3 ubiquitin ligase complex and exhibits high expression in the brain, where it regulates neuronal pathways involved in development and energy homeostasis. The identified BMI QTL on SSC1 overlaps a QTL of backfat thickness in pigs [68], consistent with pleiotropic effects linking adiposity and growth. *ZER1* was reported to be associated with LDL cholesterol levels [69], BMI [70] and anorexia nervosa [71] in humans, validating its conservative role in energy balance and body composition regulation across species.

*KLHL29* encodes a Kelch-like protein that mediates ubiquitination and protein degradation and is a strong candidate for metabolic regulation. PigGTEx phenome-wide association analysis showed that this gene was associated with circulating LDL cholesterol levels (*P* = 2.0 × 10^–11^; Additional file 3: Fig. S7) [72], consistent with a possible role in lipid homeostasis [73]. This aligns with human GWAS linking the orthologous region to lipid metabolism, obesity [74–76], and HbA1c levels [77], supporting evolutionary conservation of its function in growth and metabolism.

*HAO1* encodes hydroxyacid oxidase 1, a peroxisomal enzyme predominantly expressed in liver and proximal tubule cells, with potent activity in fatty acid and glyoxylate oxidation [78, 79]. Our identified QTL for BMI on SSC17 overlaps a liver-specific enhancer with Hi-C connections to *HAO1*, accompanied by hepatocyte-enriched expression (PigGTEx [64]) and active enhancer predictions at lead variants (*Basenji* CNN). These features establish *HAO1* as the primary effector for liver-mediated energy and lipid metabolism underlying BMI and fat deposition in pigs. Human GWAS results bolster this by linking *HAO1* to aortic stenosis risk independent of coronary artery disease (CAD) [80] and type 2 diabetes susceptibility [81]. Notably, despite the proximity of *BMP2*—a known regulator of skeletal development [82]—the strong metabolic signal and hepatic regulatory annotations prioritize *HAO1* for body composition traits. This implies a locus-specific separation of metabolic effects from skeletal growth, although minor contributions from *BMP2* cannot be fully ruled out.

Finally, for the QTL of BW and BMI on SSC2, *OR2T27* was prioritized among clustered olfactory receptor genes based on brain Hi-C data placing it in the same topologically associating domain as the lead signal. Olfactory receptors can modulate feeding behavior and energy intake [83, 84], offering a putative indirect link to BW and BMI regulation.

### Functionally informed genomic prediction and implications for breeding

Integrating candidate regulatory variants from GWAS and fine-mapping into genomic prediction models remains a key challenge for balancing biological interpretability and predictive robustness. Although Bayesian variable-selection methods (e.g., BayesB, BayesR, BayesRC) and iterated weighted GBLUP [85] alleviate over-shrinkage of major-effect loci in standard GBLUP [21], they are sensitive to prior specifications and local LD, often yielding large-effect variants—especially in high-LD regions—that lack mechanistic grounding. We addressed this by implementing a GWAS-guided linear mixed model that treats candidate functional variants as fixed effects while modeling the polygenic background as random. This separates the targeted loci from the genome-wide background, reducing over-shrinkage of these loci and allowing for an estimate their additional predictive contribution [25, 86].

In repeated cross-validation, this approach consistently improved predictive accuracy over GBLUP, with the largest gains observed for traits with well-characterized major-effect QTLs. It thus offers a scalable framework for incorporating multi-omics data to better incorporate trait-associated regulatory evidence into genomic prediction [87]. Improved prediction accuracy alone does not justify direct application in breeding programs. Persistence of major-effect alleles under intensive selection suggests maintenance by antagonistic pleiotropy and balancing selection [88], whereby production benefits may be offset by costs to unmeasured fitness or robustness traits. Our results therefore constitute a methodological advance rather than a selection directive; practical use demands integration into multi-trait indices with monitoring of correlated responses to preserve long-term population resilience.

## Limitations and future directions

Although this study uses multi-omics and computational methodologies to understand the regulatory architecture of BW and BMI, more detailed mapping is required to fully build the underlying regulatory networks. Future efforts should leverage single-cell multi-omics, such as single-cell RNA sequencing (scRNA-seq) and single-cell assay for transposase-accessible chromatin using sequencing (scATAC-seq), to resolve cell-type-specific regulatory mechanisms that remain obscured in bulk tissue data [89, 90]. Furthermore, while we prioritize *ZER1*, *KLHL29*, *HAO1*, and *OR2T27* as putative target genes, establishing definitive causality will require experimental validation. Targeted approaches, including CRISPR-Cas9-mediated allelic editing and tissue-specific perturbations [91], will be essential to mechanistically link these candidates to gene regulation and phenotypic variation in body size and metabolism.

## Conclusion

Our study provides some insights into the regulatory mechanisms of BW and BMI, suggesting that candidate target genes may connect neural signaling with metabolic homeostasis. The improved genomic prediction performance obtained using these prioritized variants supports their association with BW and BMI. These findings not only refine our understanding of complex trait regulation but also underscore the critical value of integrating multi-omics data to resolve functional candidates in the post-GWAS era.

## Supplementary Information

Additional file1 (XLSX 14 KB): Fig. S1. Candidate functional SNPs of BW and BMI.

Additional file2 (XLSX 3854 KB): Fig. S2. Gene-based association studies (MAGMA) of BW and BMI.

Additional file3 (PDF 19 KB): Fig. S3. Linkage disequilibrium decay in the Shanxia Long Black pig. x- and y-axes are the physical distance and *r*^2^ between SNPs, respectively.

Additional file4 (PDF 877 KB): Fig. S4. The distributions of adjusted BW and BMI. a, BW. b, BMI. The figure shows the frequency (rectangles) and density (curve line) after normalization and correction.

Additional file5 (PDF 6738 KB): Fig. S5. Genomic regions associated with BW and BMI. (a), with BW from 52,429,964 to 53,429,964 on SSC2. (b), with BW from 63,359,520 to 64,359,520 on SSC2. c, with BW from 115,890,779 to 116,890,779 on SSC3. (d), with BW from 1,966,231 to 2,966,231 on SSC7. (e), with BW from 27,082,460 to 28,082,460 on SSC11. (f), with BW from 22,266,783 to 23,266,783 on SSC12. (g), with BMI from 52,327,455 to 53,327,455 on SSC2. In each plot, the top panel shows the negative logarithms (base 10) of *P* values and linkage disequilibrium degrees (*r*^2^) to the top SNP, and the bottom panel shows the annotated genes in the region.

Additional file6 (PDF 1842 KB): Fig. S6. Chromatin state annotation, Hi-C, and in silico mutagenesis of the BW locus on SSC2. (a), Chromatin state landscapes of 14 porcine tissues at the locus of BW in the region from 52,838,612 to 52,943,942. The tracks depict 15 distinct chromatin states, categorizing functional elements into active promoters (TssA, TssAHet, and TssBiv), TSS-proximal transcribed regions (TxFlnk, TxFlnkWk, and TxFlnkHet), active and poised enhancers (EnhA, EnhAMe, EnhAWk, EnhAHet, and EnhPois), repressed regions (Repr, and ReprWk), and quiescent chromatin (Qui). Accessible chromatin peaks are marked as ATAC islands. (b), Hi-C contact maps at 5-kb resolution from brain tissue, highlighting TAD and chromatin loop structures. (c), In silico saturation mutagenesis analysis of the candidate variant SSC2_52929964 in the cerebellum.

Additional file7 (PDF 890 KB): Fig. S7. AUROC and AUPRC performance of *Basenji* models trained on adipose, liver, and brain tissues. (a), AUROC boxplots. (b), AUPRC boxplots.

Additional file8 (PDF 236 KB): Fig. S8. *HAO1* expression levels (TPM) across tissues and cell types from PigGTEx. (a), Bulk RNA-seq-derived tissue-level expression of HAO1. (b), Single-cell RNA-seq-based cell-type-specific expression from the PigGTEx atlas.

Additional file9 (PDF 1155 KB): Fig. S9. Results of henome-wide association analysis (PheWAS) for the KLHL29 (*ENSSSCG00000008593*) gene.

## Data Availability

The data that support the findings of this study are available from the corresponding author upon reasonable request.
