# Supplementary material for: Functional genomic dissection and prediction of body size traits in pigs
Source: Genet Sel Evol. 2026 Aug 16;58:55. doi: 10.1186/s12711-026-01078-1 (PMC13501698; doi:10.1186/s12711-026-01078-1)
Supplement: Supplementary file 1 [file 12711_2026_1078_MOESM3_ESM.pdf]

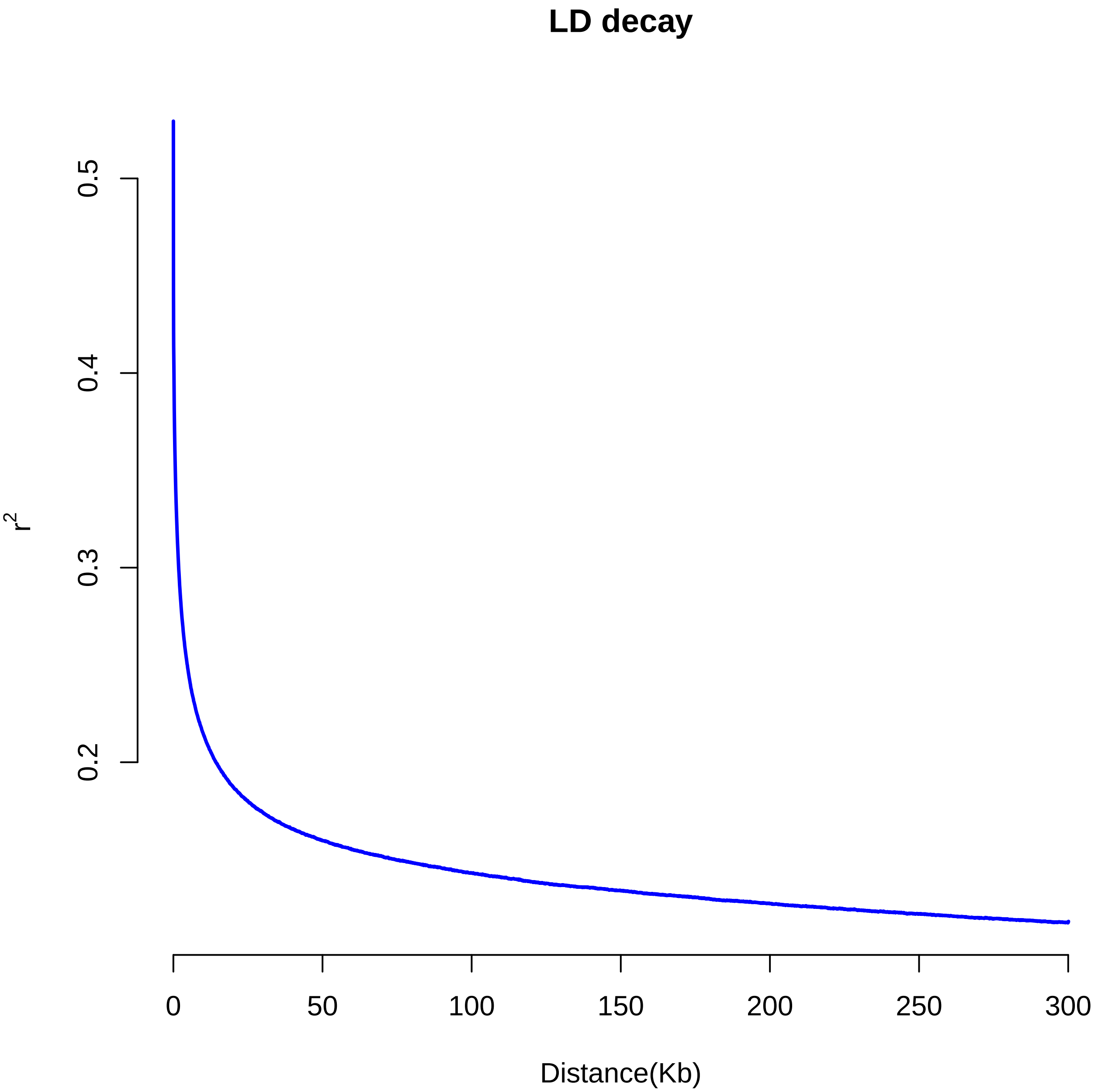
LD decay
0.5
0.4
0.3
0.2
r2
0
50
100
150
200
250
300
Distance(Kb)
