# Supplementary material for: Functional genomic dissection and prediction of body size traits in pigs
Source: Genet Sel Evol. 2026 Aug 16;58:55. doi: 10.1186/s12711-026-01078-1 (PMC13501698; doi:10.1186/s12711-026-01078-1)
Supplement: Supplementary file 2 [file 12711_2026_1078_MOESM4_ESM.pdf]

a

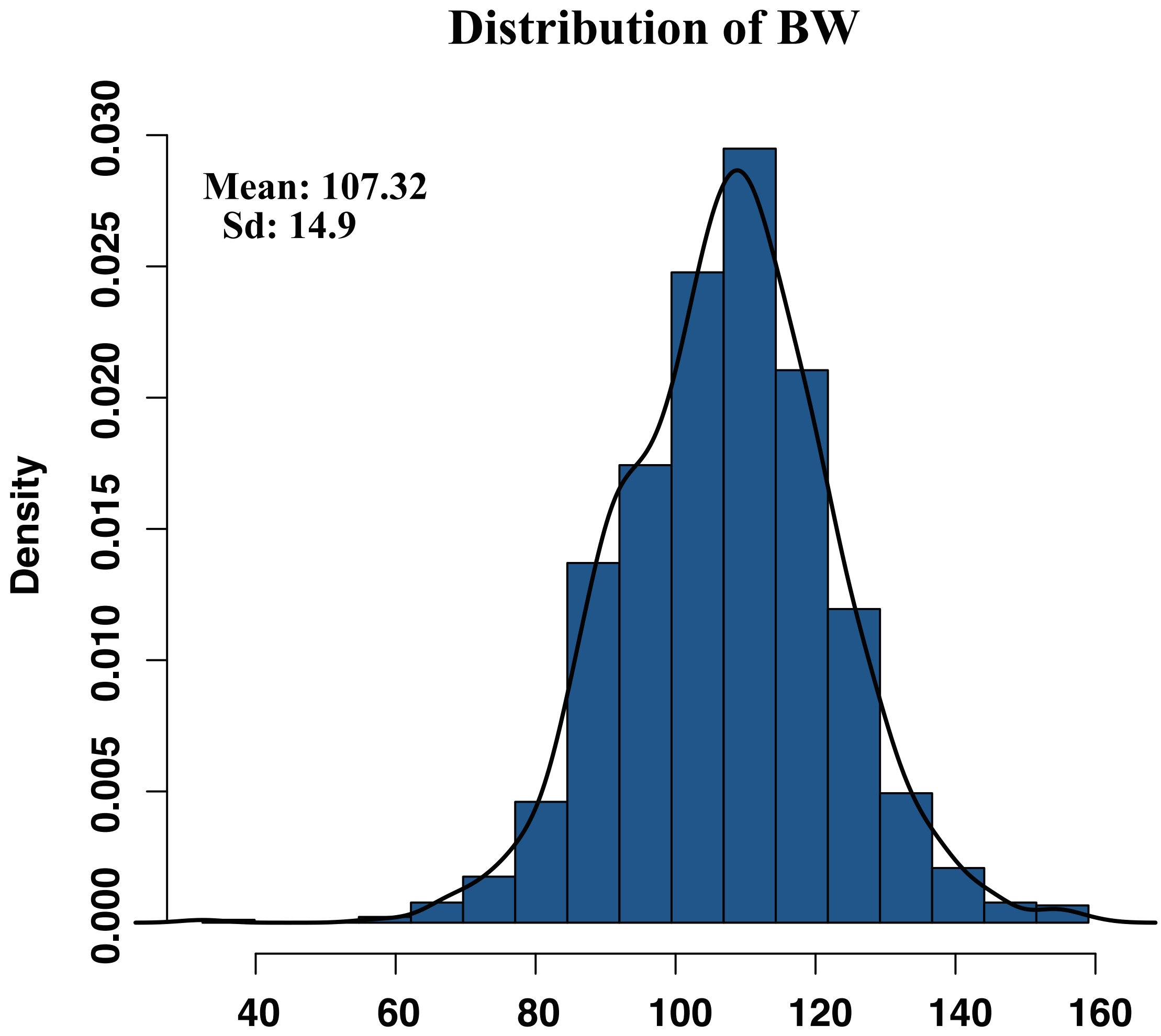
Distribution of BW
Mean: 107.32
Sd: 14.9
Density
0.000
0.005
0.010
0.015
0.020
0.025
0.030
40
60
80
100
120
140
160

b

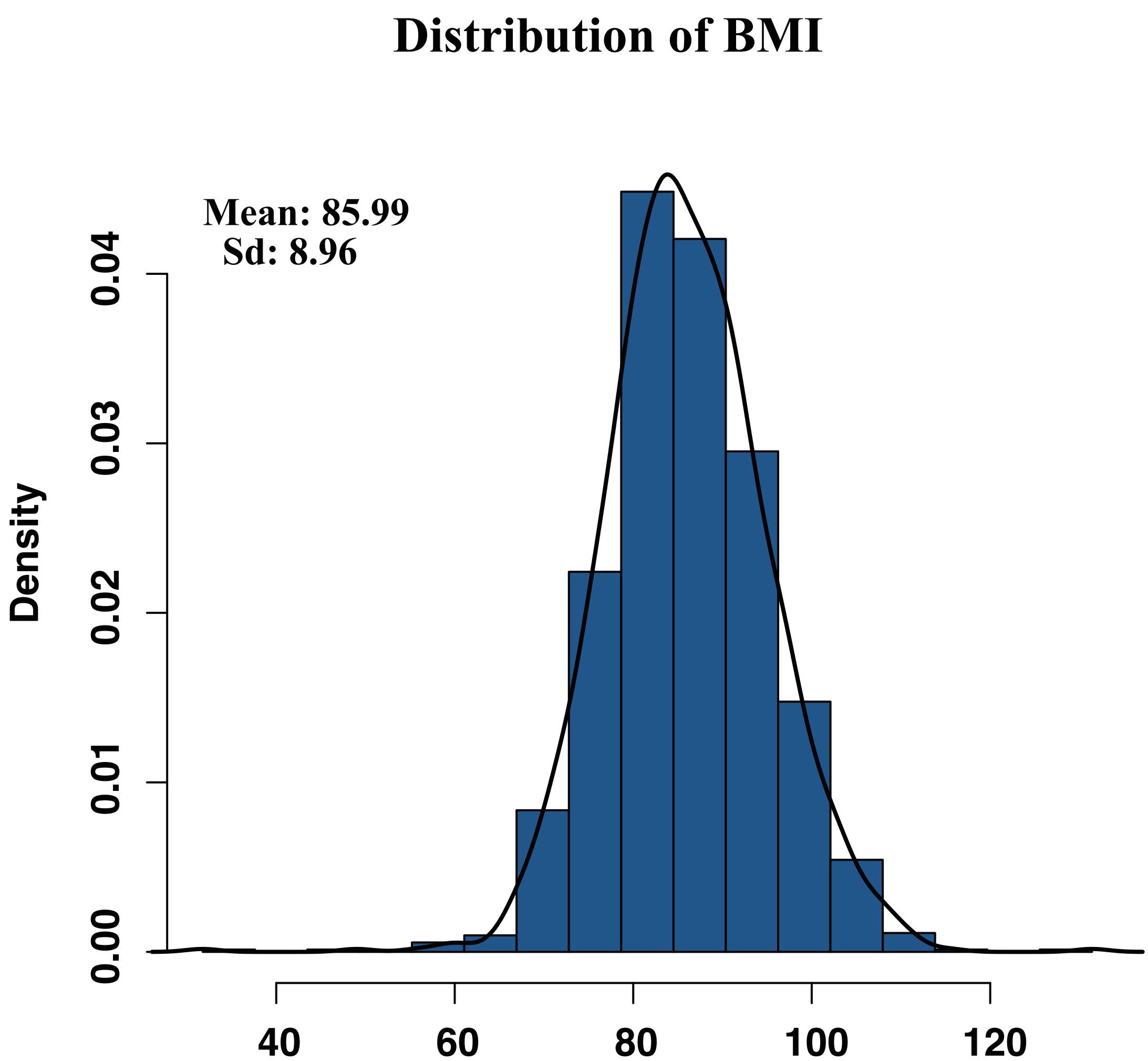
Distribution of BMI
Mean: 85.99
Sd: 8.96
Density
0.00
0.01
0.02
0.03
0.04
40
60
80
100
120
