# Supplementary material for: Functional genomic dissection and prediction of body size traits in pigs
Source: Genet Sel Evol. 2026 Aug 16;58:55. doi: 10.1186/s12711-026-01078-1 (PMC13501698; doi:10.1186/s12711-026-01078-1)
Supplement: Supplementary file 3 [file 12711_2026_1078_MOESM5_ESM.pdf]

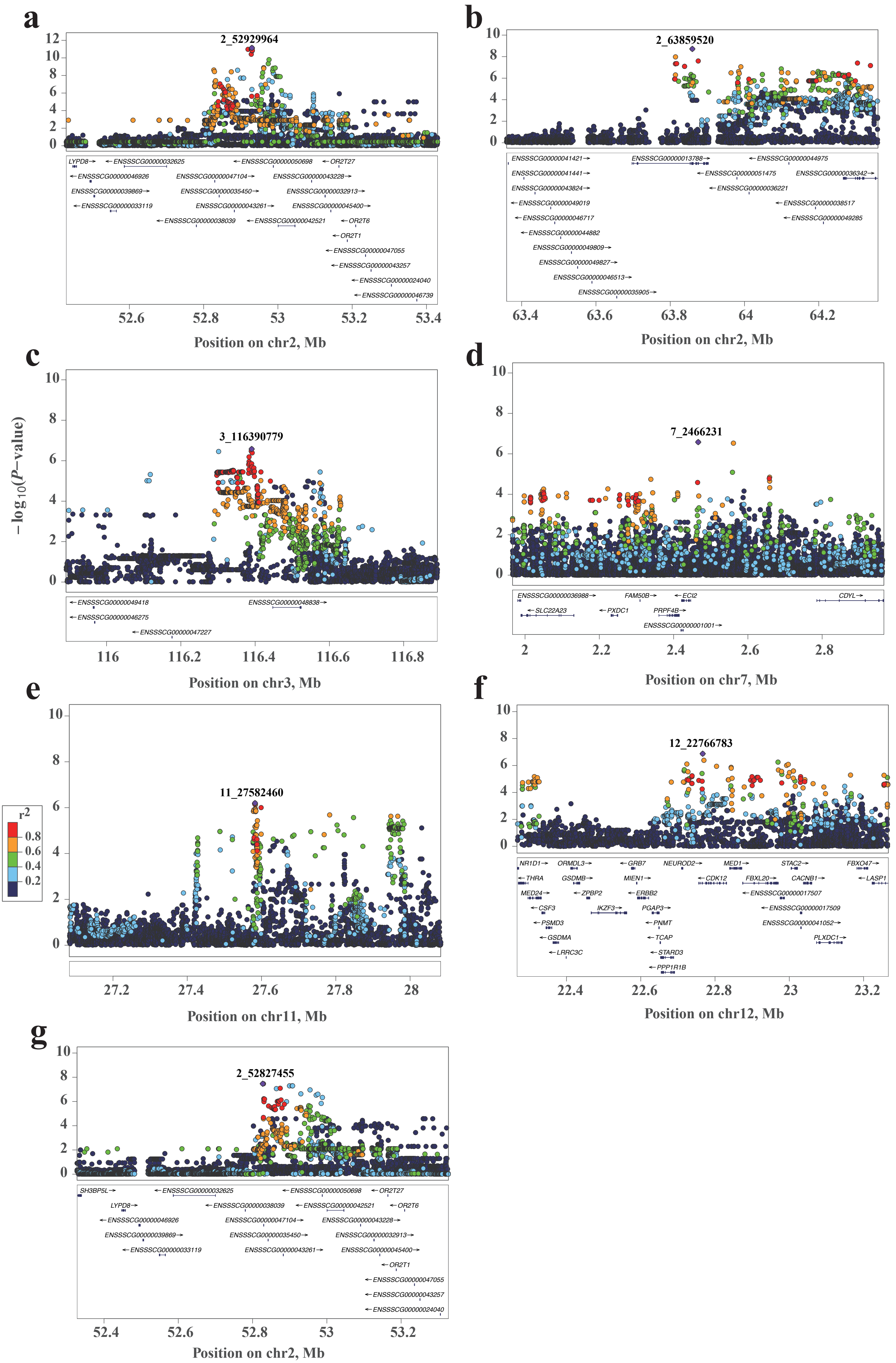

a
2_52929964
LYPD8
ENSSSCG00000032625
ENSSSCG00000050698
OR2T27
ENSSSCG00000046926
ENSSSCG00000047104
ENSSSCG00000043228
ENSSSCG00000039869
ENSSSCG00000035450
ENSSSCG00000032913
ENSSSCG00000033119
ENSSSCG00000043261
ENSSSCG00000045400
ENSSSCG00000038039
ENSSSCG00000042521
OR2T6
OR2T1
ENSSSCG00000047055
ENSSSCG00000043257
ENSSSCG00000024040
ENSSSCG00000046739
Position on chr2, Mb
b
2_63859520
ENSSSCG00000041421
ENSSSCG00000013788
ENSSSCG00000044975
ENSSSCG00000041441
ENSSSCG00000051475
ENSSSCG00000036342
ENSSSCG00000043824
ENSSSCG00000036221
ENSSSCG00000049019
ENSSSCG00000038517
ENSSSCG00000046717
ENSSSCG00000049285
ENSSSCG00000044882
ENSSSCG00000049809
ENSSSCG00000049827
ENSSSCG00000046513
ENSSSCG00000035905
Position on chr2, Mb
c
−log10(P−value)
3_116390779
ENSSSCG00000049418
ENSSSCG00000048838
ENSSSCG00000046275
ENSSSCG00000047227
Position on chr3, Mb
d
7_2466231
ENSSSCG00000036988
FAM50B
ECI2
CDYL
SLC22A23
PXDC1
PRPF4B
ENSSSCG00000001001
Position on chr7, Mb
e
11_27582460
Position on chr11, Mb
r2
0.8
0.6
0.4
0.2
f
12_22766783
NR1D1
ORMDL3
GRB7
NEUROD2
MED1
STAC2
FBXO47
THRA
GSDMB
MIEN1
CDK12
FBXL20
CACNB1
LASP1
MED24
ZPBP2
ERBB2
ENSSSCG00000017507
CSF3
IKZF3
PGAP3
ENSSSCG00000017509
PSMD3
PNMT
ENSSSCG00000041052
GSDMA
TCAP
PLXDC1
LRRC3C
STARD3
PPP1R1B
Position on chr12, Mb
g
2_52827455
SH3BP5L
ENSSSCG00000032625
ENSSSCG00000050698
OR2T27
LYPD8
ENSSSCG00000038039
ENSSSCG00000042521
OR2T6
ENSSSCG00000046926
ENSSSCG00000047104
ENSSSCG00000043228
ENSSSCG00000039869
ENSSSCG00000035450
ENSSSCG00000032913
ENSSSCG00000033119
ENSSSCG00000043261
ENSSSCG00000045400
OR2T1
ENSSSCG00000047055
ENSSSCG00000043257
ENSSSCG00000024040
Position on chr2, Mb
