# Supplementary material for: Functional genomic dissection and prediction of body size traits in pigs
Source: Genet Sel Evol. 2026 Aug 16;58:55. doi: 10.1186/s12711-026-01078-1 (PMC13501698; doi:10.1186/s12711-026-01078-1)
Supplement: Supplementary file 4 [file 12711_2026_1078_MOESM6_ESM.pdf]

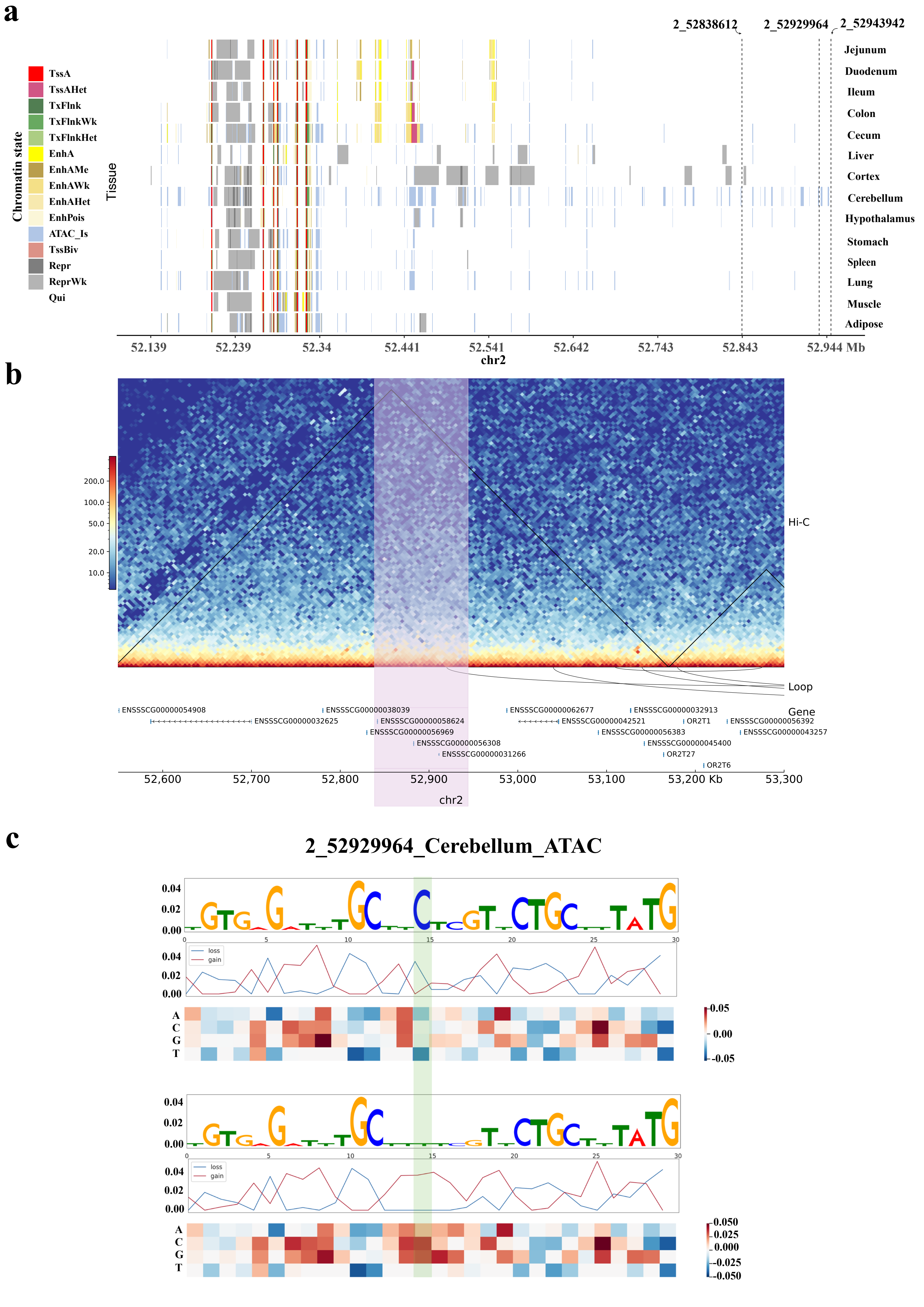

a
2_52838612
2_52929964
2_52943942
Chromatin state
TssA
TssAHet
TxFlnk
TxFlnkWk
TxFlnkHet
EnhA
EnhAMe
EnhAWk
EnhAHet
EnhPois
ATAC_Is
TssBiv
Repr
ReprWk
Qui
Tissue
Jejunum
Duodenum
Ileum
Colon
Cecum
Liver
Cortex
Cerebellum
Hypothalamus
Stomach
Spleen
Lung
Muscle
Adipose
52.139
52.239
52.34
52.441
52.541
52.642
52.743
52.843
52.944 Mb
chr2
b
200.0
100.0
50.0
20.0
10.0
Hi-C
Loop
Gene
ENSSSCG00000054908
ENSSSCG00000032625
ENSSSCG00000038039
ENSSSCG00000058624
ENSSSCG00000056969
ENSSSCG00000056308
ENSSSCG00000031266
ENSSSCG00000062677
ENSSSCG00000042521
ENSSSCG00000032913
OR2T1
ENSSSCG00000056392
ENSSSCG00000056383
ENSSSCG00000043257
ENSSSCG00000045400
OR2T27
OR2T6
52,600
52,700
52,800
52,900
53,000
53,100
53,200 Kb
53,300
chr2
c
2_52929964_Cerebellum_ATAC
loss
gain
A
C
G
T
0.05
0.00
-0.05
0.050
0.025
0.000
-0.025
-0.050
