# Supplementary material for: Functional genomic dissection and prediction of body size traits in pigs
Source: Genet Sel Evol. 2026 Aug 16;58:55. doi: 10.1186/s12711-026-01078-1 (PMC13501698; doi:10.1186/s12711-026-01078-1)
Supplement: Supplementary file 5 [file 12711_2026_1078_MOESM7_ESM.pdf]

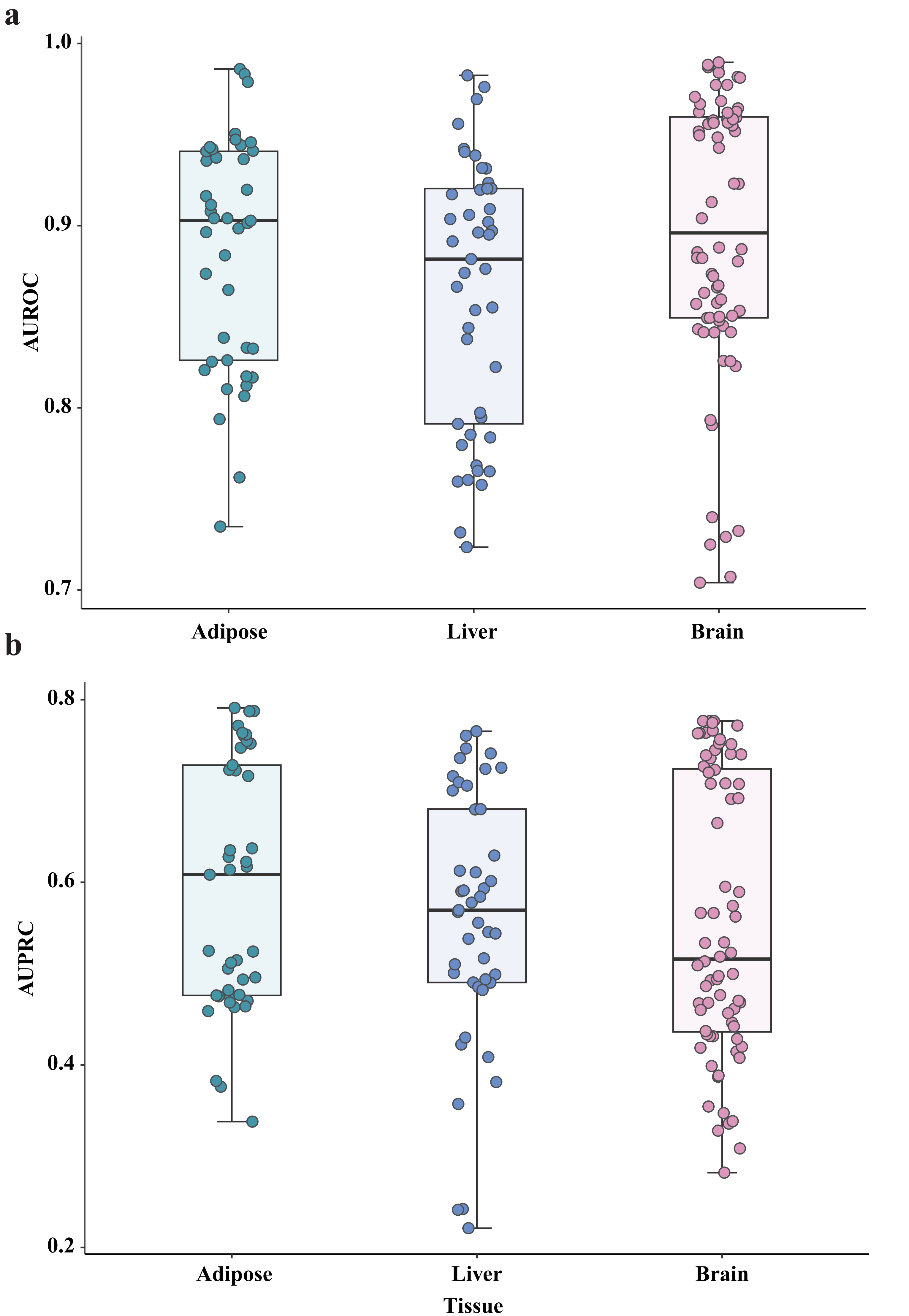
a
1.0
0.9
0.8
0.7
AUROC
Adipose
Liver
Brain
b
0.8
0.6
0.4
0.2
AUPRC
Adipose
Liver
Brain
Tissue
