# Supplementary material for: Functional genomic dissection and prediction of body size traits in pigs
Source: Genet Sel Evol. 2026 Aug 16;58:55. doi: 10.1186/s12711-026-01078-1 (PMC13501698; doi:10.1186/s12711-026-01078-1)
Supplement: Supplementary file 6 [file 12711_2026_1078_MOESM8_ESM.pdf]

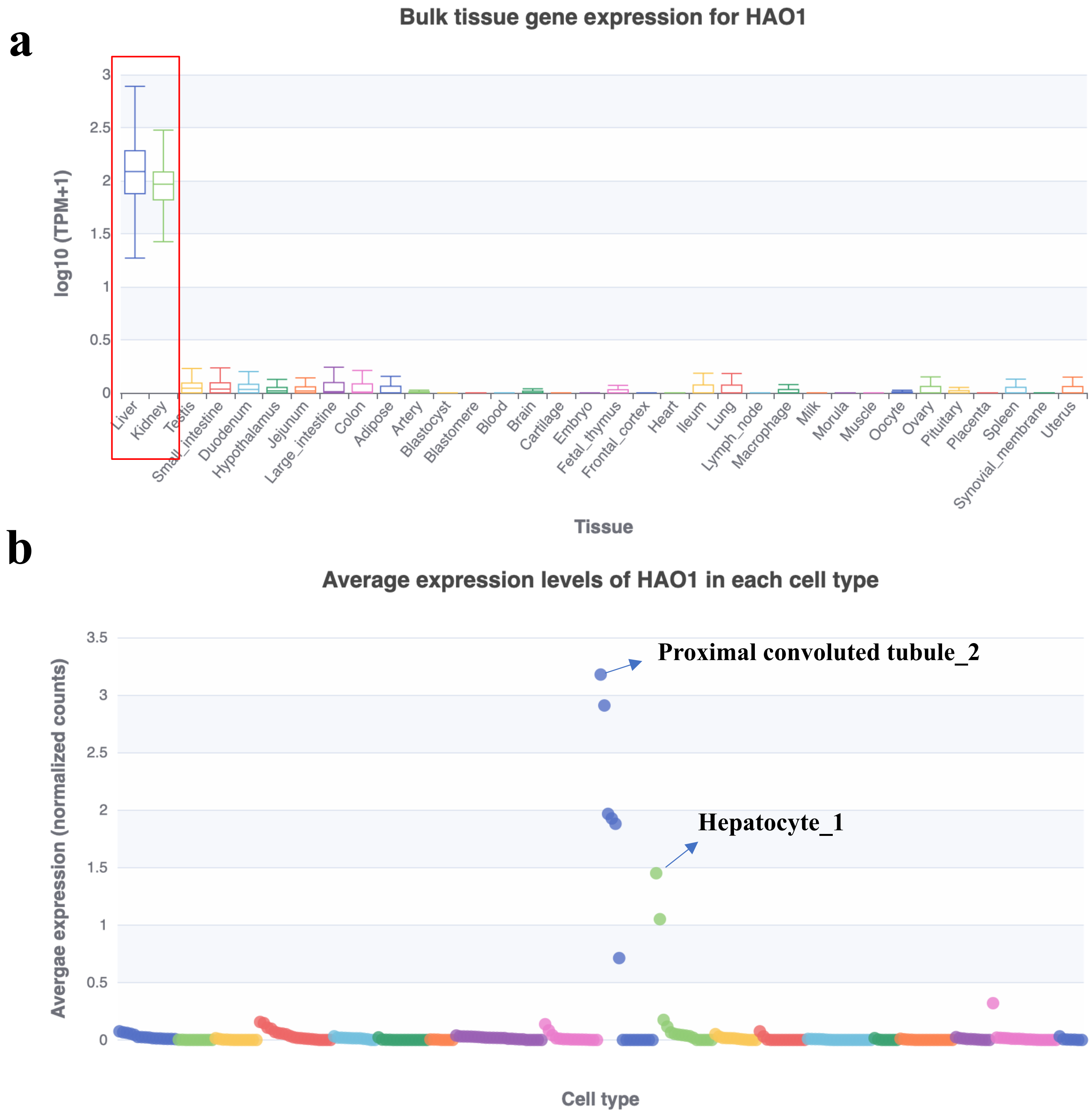
a
Bulk tissue gene expression for HAO1
log10 (TPM+1)
3
2.5
2
1.5
1
0.5
0
Liver
Kidney
Testis
Small_intestine
Duodenum
Hypothalamus
Jejunum
Large_intestine
Colon
Adipose
Artery
Blastocyst
Blastomere
Blood
Brain
Cartilage
Embryo
Fetal_thymus
Frontal_cortex
Heart
Ileum
Lung
Lymph_node
Macrophage
Milk
Morula
Muscle
Oocyte
Ovary
Pituitary
Placenta
Spleen
Synovial_membrane
Uterus
Tissue
b
Average expression levels of HAO1 in each cell type
Avergae expression (normalized counts)
3.5
3
2.5
2
1.5
1
0.5
0
Proximal convoluted tubule_2
Hepatocyte_1
Cell type
