# Supplementary material for: Functional genomic dissection and prediction of body size traits in pigs
Source: Genet Sel Evol. 2026 Aug 16;58:55. doi: 10.1186/s12711-026-01078-1 (PMC13501698; doi:10.1186/s12711-026-01078-1)

PheWAS of ENSSSCG00000008593

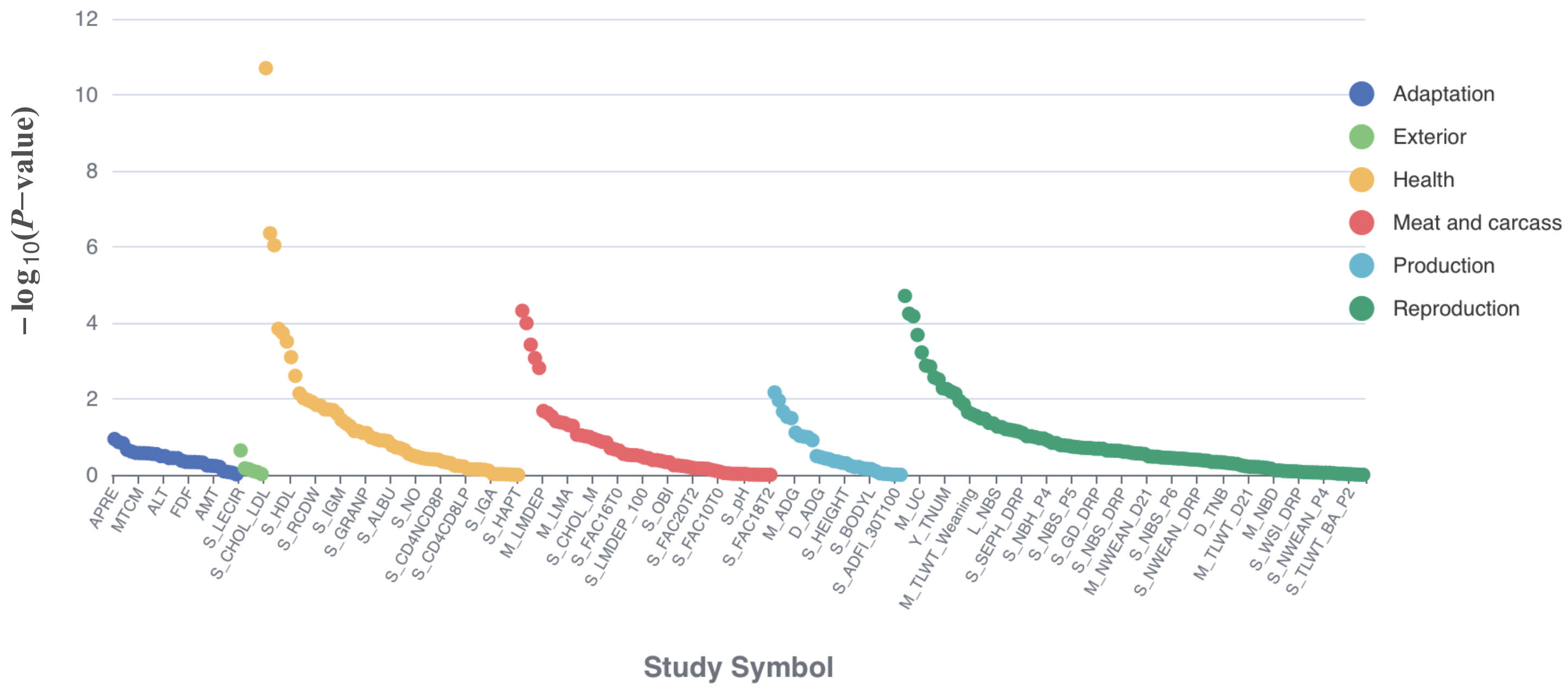

Supplement: Supplementary file 7 [file 12711_2026_1078_MOESM9_ESM.pdf]
